# Human color constancy based on the geometry of color distributions

**DOI:** 10.1167/jov.21.3.7

**Published:** 2021-03-04

**Authors:** Takuma Morimoto, Takahiro Kusuyama, Kazuho Fukuda, Keiji Uchikawa

**Affiliations:** 1 Department of Experimental Psychology, University of Oxford, Oxford, UK; 2 Department of Information Processing, Tokyo Institute of Technology, Yokohama, Japan; 3 Department of Information Design, Kogakuin University, Tokyo, Japan; 4 Human Media Research Center, Kanagawa Institute of Technology, Atsugi, Japan

**Keywords:** color vision, color constancy, optimal color, illuminant estimation

## Abstract

The physical inputs to our visual system are dictated by the interplay between lights and surfaces; thus, for surface color to be stably perceived, the influence of the illuminant must be discounted. To reveal our strategy to infer the illuminant color, we conducted three psychophysical experiments designed to test our optimal color hypothesis that we internalize the physical color gamut under various illuminants and apply the prior to estimate the illuminant color. In each experiment, we presented 61 hexagons arranged without spatial gaps, where the surrounding 60 hexagons were set to have a specific shape in their color distribution. We asked participants to adjust the color of a center test field so that it appeared to be a full-white surface placed under a test illuminant. Results and computational modeling suggested that, although our proposed model is limited in accounting for estimation of illuminant intensity by human observers, it agrees fairly well with the estimates of illuminant chromaticity in most tested conditions. The accuracy of estimation generally outperformed other tested conventional color constancy models. These results support the hypothesis that our visual system can utilize the geometry of scene color distribution to achieve color constancy.

## Introduction

The cone excitations associated with an illuminated surface is determined both by the spectral composition of illuminant and by the surface spectral reflectance; thus, when a scene illuminant changes, the associated cone signals accordingly change. However, numerous tests of human perception of colored surfaces indicate a high level of perceptual constancy, in which the appearance of the surface is relatively little changed (Foster, 2011). The degree to which surface color is stably perceived is often referred to as color constancy, which enables us to identify objects by their color in spite of the change of illumination. However, the exact mechanisms underpinning this stability of the color vision system are not fully understood yet.

One common way to conceptualize color constancy is that the visual system first estimates the color of scene illumination based on cues available in the scene and then discounts its influence from the whole scene. Past color constancy research has been successful in identifying a number of mechanisms thought to underlie color constancy (Smithson, 2005; Hurlbert, 2007). In terms of neural substrates, both local (von Kries 1905; Smithson & Zaidi, 2004) and global (Ives, 1912; Werner, 2014) retinal adaptation is known to be useful in the implementation of color constancy. Identifying statistics-based cues to the illuminant has been a primary focus in behavioral color constancy studies. Perhaps the simplest method would be to find the brightest surface in the scene. This is based on the observation that a white surface with a completely flat spectral reflectance reflects any lights in a spectrally unmodified way, meaning that it conveys direct information about the color of the illumination (Land, 1977). Also, Tominaga, Ebisui, and Wandell (2001) suggested from a computational point of view that brighter surfaces are better cues than darker surfaces. Another simple but powerful transformation would be to compute mean chromaticity across all surfaces in a scene and assume that it is a good estimate of the illuminant color (Buchsbaum, 1980). This algorithm stands on the idea that the average color across all objects in a scene is typically gray (gray-world hypothesis), and thus the deviation from gray can be assumed to be due to the influence of the illumination.

It is also known that spatial mean cone signals, rather than mean chromaticity, would be a good predictor of an illuminant color (Khang & Zaidi, 2004). The use of these simple global statistics is typically relevant only to scenes that are uniformly illuminated and are also particularly effective only when the scene contains many different surface spectral reflectances. Foster and Nascimento (1994) found that the cone ratio between neighboring surfaces in natural scenes tends to be constant under the change of illuminants, allowing our visual system to discriminate illuminant color change and surface color change by spatial comparison of cone signals. It is worth noting that identifying cues that are in theory useful does not guarantee that our visual system can or does use them. It is therefore important to experimentally verify that candidate cues are actually used by human observers. Moreover, it is good to remind ourselves that we may not rely on a single specific mechanism but rather may use a combination of various cues. Such a strategy is useful in realistic situations, as a specific cue may not be available in every scene (Kraft & Brainard, 1999).

Using global scene statistics (e.g., mean chromaticity) to estimate the color of illumination has an attractive simplicity. However, although they reasonably account for experimental data, one of the longstanding questions in the field of human color vision has been how our visual system distinguishes a white scene illuminated by a reddish illumination from a reddish scene illuminated by a whitish illumination when both cases produce the same reddish mean chromaticity (Brown, 1994). If our visual system relies purely on a chromaticity-based solution, the estimation of an illuminant color in both cases should correspond to the chromaticity of the spatial mean (i.e., red); thus, a reddish scene should incorrectly appear as a white scene. Golz and MacLeod (2002) provided a solution to this question. First, they analyzed hyperspectral natural images and found that L/(L+M) and luminance are positively correlated when the illumination is reddish. In contrast, when the illumination is white, this chromaticity–luminance correlation is not observed. Therefore, by looking at the correlation between chromaticity and luminance across scene surfaces in an individual scene, it is possible to work out the color of the illumination lighting the scene. Second, importantly, they showed psychophysically that human observers are able to use this cue to solve such an ambiguity in a biased surface color set (e.g., dominantly reddish surfaces). We note that one study found that this observation regarding chromaticity–luminance correlation might be limited to scenes including foliage (Ciurea & Funt, 2004). There is also debate about whether the effect of the luminance–redness correlation on color appearance is spatially local (Granzier, Brenner, Cornelissen, & Smeets, 2005) or globally held (Golz, 2008). In any case, it is implied that the way in which luminances distribute over various chromaticities seems to play a role in the implementation of color constancy, particularly in scenes that are chromatically biased.

Statistical models such as Bayesian estimation (Brainard & Freeman, 1997; Brainard, Longère, Delahunt, Freeman, Kraft, & Xiao, 2006) have been reported to be good candidate models of human color constancy. Also, Maloney and Wandell (1986) proposed that it is possible to recover surface spectral reflectance of objects in a scene where spectral functions of surface reflectance and illuminant can be determined by a linear-weighted sum of a small number of basis vectors. Interestingly, these theoretical models make use of statistical regularities of surface spectral reflectance and illuminant spectra that occur in the natural environment, implying that the human visual system utilizes such constraints. This also makes sense from a mathematical point of view, as in general ill-posed problems can be solved when we put in a sufficient number of assumptions.

Moreover, the study of computational color constancy has been an active domain in the field of computer vision. A vast number of methods based on simple image statistics have been proposed (e.g., summarized by Barnard, Cardei, & Funt, 2002; Arjan, Theo, & van de Weijer, 2011), including gray-world and white-patch (“brightest is white”) algorithms, which we introduce as candidate mechanisms in human color constancy. Gamut mapping is a unique approach based on the observation that only limited colors are observed under a given illuminant (Forsyth, 1990; Finlayson, 1996). This range of a limited colors is termed the *canonical gamut*. This canonical gamut must be learned from a training set that represents colors that can occur in the test images. The model aims to find the computational mapping so that the gamut of the input image is mapped to the canonical gamut for a given illuminant, thereby allowing the illuminant influence to be discounted. In more recent years, the color-by-correlation method has further developed the gamut mapping method to a more general framework by substituting a correlation matrix for the canonical gamut (Finlayson, Hordley, & Hubel, 2001). Barron (2015) suggested a framework termed *convolutional color constancy* (CCC) that operates based on a histogram of chrominance (which is essentially equivalent to chromaticity). The basic idea is to use the observation that, in a logarithmic chrominance space, the shape of the histogram is nearly invariant under the change of illuminant color, and the entire histogram shifts toward the color of illuminant. The two-dimensional (2D) chrominance histogram of an input image is convolved with a filter that is optimized in a prior training process using separate training-set chrominance histograms and ground-truth illuminations so that the optimized filter generates the strongest response at the ground-truth illuminant when convolved with the histogram of an input image. The convolved 2D map provides a posterior probability for the color of illuminant in an input image.

Fast Fourier Color Constancy, introduced later, also works on fundamentally the same basis, but it is a significant improvement over CCC in terms of computation time (Barron & Tsai, 2017). A method for illuminant estimation using a neural network was proposed in an early study (Funt, Cardei, & Barnard, 1996), and, in more recent years, it has been rapidly expanded due to advancements made in machine learning tools. For example, illuminant estimation using convolutional neural networks (CNNs) (Bianco, Cusano, & Schettini, 2015; Choi, Kang, & Yun, 2020) and generative adversarial network-based models has been proposed (Das, Baslamisli, Liu, Karaoglu, & Gevers, 2018). These neural-network-based methods contrast with past algorithm-based approaches because the sequence of steps to estimate the illuminant color does not have to be explicitly defined. Instead, networks are trained by a large number of training image datasets, and the network automatically extracts image features that allows the network to estimate the illuminant color from a new test image. The training typically requires tuning of millions of parameters, and the trained network is often difficult to interpret. Whether such complexity is necessary to solve color constancy is still an open question (e.g., argued in Finlayson, 2018), but state-of-the-art performance has been reported for these models. Also, applying CNNs to study human color constancy has attracted increasing attention (Flachot & Gegenfurtner, 2018; Flachot, Schuett, Fleming, Wichmann, & Gegenfurtner, 2019).

Natural scenes that we see in daily life appear to contain a wide variety of color; thus, it is tempting to think that any color can exist in a scene, but this is a false intuition. In any scene, the possible range of chromaticity and luminance is limited by the spectral composition of the illuminant, in a similar way that a digital monitor has a limited color gamut. This gamut for surface colors under a particular illuminant can be visualized by optimal colors or, more precisely, *optimal surfaces* (MacAdam, 1935a; MacAdam, 1935b). Optimal color is defined as a surface that has only 0% and 100 % reflectances and has at most two abrupt spectral transitions between them. We can think of two types of optimal colors: one boots up at wavelength λ_1_ and boots off at λ_2_ (band-pass type), and the other boots off and up at λ_1_ and at λ_2_ (band-stop type), where λ_1_ < λ_2_. To give an example how optimal colors distribute and how that changes depending on the color temperature of illuminants, we prepared 102,721 optimal colors by changing λ_1_ from 400 nm to 720 nm with 1 nm and changing λ_2_ from λ_1_ to 720 nm with 1 nm for the two types of optimal colors (Uchikawa, Fukuda, Kitazawa, & MacLeod, 2012).

**Figure 1.**
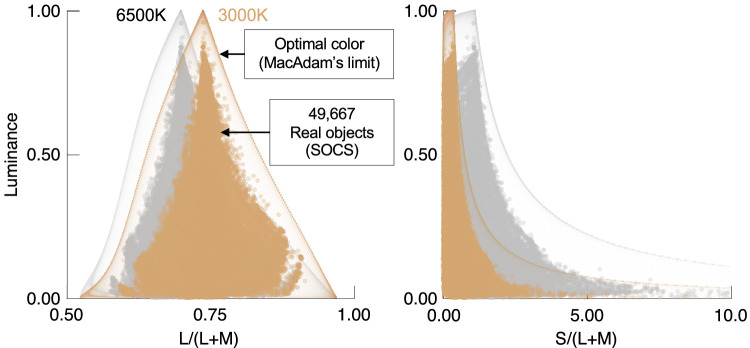
Color distribution of 102,721 optimal colors and 49,667 real objects in the SOCS database. They are rendered under 6500 K (gray distribution) and 3000 K (orange distributions) to show the effect of illuminant change. The left and right panels show L/(L+M) versus. luminance and S/(L+M) versus luminance distributions, respectively.

Figure 1 shows a distribution of 102,721 optimal colors (also known as MacAdam's limit) and 49,667 objects in the real world drawn from the standard object color spectra database for color reproduction evaluation (SOCS; ISO/TR 16066:2003) under the illuminants of 3000 K and 6500 K. The left panel shows L/(L+M) in a MacLeod–Boynton (MB) chromaticity diagram (MacLeod & Boynton, 1979) versus luminance distribution, and the right panel shows S/(L+M) versus luminance distribution. For the calculation of cone excitations, we used the Stockman and Sharpe cone fundamentals (Stockman & Sharpe, 2000). Optimal colors do not exist in the real world, but they are a useful mathematical tool to allow us to see the upper boundary of surface colors under a particular illuminant. If we look at this from the other side, under a particular illuminant any chromaticity has a unique corresponding optimal color, and the luminance of the optimal color gives the theoretical upper limit at the chromaticity.

The peak of optimal color distributions always corresponds to a full-white surface (1.0 reflectance across all wavelengths), thus providing the chromaticity and intensity of the illuminant itself (i.e., white point). Optimal colors with higher purity have lower luminance; because they have a narrower band reflectance, the luminance distribution spreads out as the purity increases. We also see that the distribution of real objects is unsurprisingly fully included within the optimal color shell, but importantly the shape looks like the optimal color distribution under both illuminants. To put it in another way, there is a strong association between the illuminant color and the way a set of colors is distributed in the real world.

**Figure 2.**
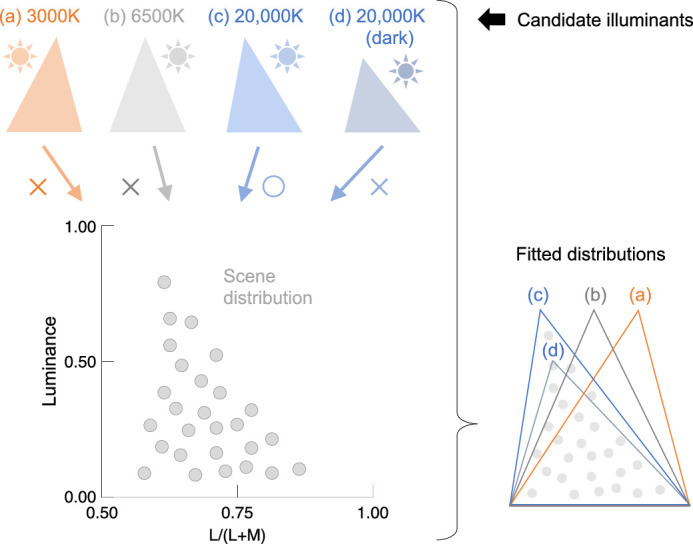
Schematic illustration of optimal color hypothesis. Our visual system internalizes optimal color distributions and selects the one that gives a most likely fit to the scene distribution. In this case, 20,000 K should be selected.

This observation led to an idea: If our visual system is aware of this imposed statistical constraint (in other words, if our visual system internalizes the shape of optimal color distribution under various illuminants), then we can inversely work out from the observed chromaticity versus luminance distribution to compute the influence of illuminant color (Uchikawa et al., 2012). The simplest algorithm to implement this concept would be to find the best-fit optimal color shell for a given color distribution of a scene. This idea is depicted in Figure 2. For a given scene distribution, we fit the optimal color distribution of candidate illuminants—in this case, (a) 3000 K, (b) 6500 K, (c) 20,000 K, and (d) darker 20,000 K. For this example, the 20,000 K illuminant fits the scene distribution best. The 3000 K and 6500 K illuminants are not appropriate, as some surfaces exceed the optimal color distributions. Also, the darker 20,000 K illuminant does not hold some surfaces because the luminance level is too low, even though the color temperature is the same as the 20,000 K illuminant. This highlights the importance of selecting an appropriate illuminant intensity, as well as color temperature, in the framework of the optimal color model. Such an algorithm should function perfectly when a scene contains only optimal colors, because we can uniquely find the optimal color distribution that fits to the scene distribution perfectly. However, in more general situations, where a scene does not contain optimal colors, illuminant estimation is more challenging, as we need to find the most appropriate optimal color distribution—for example, by minimizing the root-mean-square error between optimal color distribution and scene distribution.

In summary, our hypothesis based on the optimal color model is as follows: When the visual system observes a scene, light sources and specular highlights have to be rejected first. Then, based on the remaining surface colors, our visual system seeks the most likely optimal color distribution such that it covers all surface colors in a scene.

It might be worth noting that our model has some parallels with the gamut mapping method developed for computational color constancy in terms of utilizing an observed range of colors in a scene, but it also differs in the following ways. The major difference is the use of optimal colors as an internal template. Our model assumes that the human visual system learns the physical envelope of surface colors (i.e., optimal colors distributions) through observation of the rich amount of surface colors under various illuminants in daily life and, importantly, how this distribution changes in response to illuminant color changes. In contrast, the gamut mapping method uses the possible range of RGB values that can be observed only under a canonical illuminant (i.e., canonical gamut) as an internal template. Also, computation of our model predictions is performed in an opponent color space in which chromaticity and luminance are explicitly separated and independent, instead of in RGB color space. Thus, the way the chromaticities of scene surfaces are associated with luminance provides a key cue to our model, paralleling the suggestion of luminance–color correlation mechanisms for human color constancy (Golz & MacLeod, 2002).

In a series of previous papers, we have demonstrated the importance of the luminance–chromaticity association (Uchikawa et al., 2012; Morimoto, Fukuda, & Uchikawa, 2016). It was also shown that observers seem to ignore surfaces that appear self-luminous from the consideration of illuminant influence (Fukuda & Uchikawa, 2014). We also suggested that the optimal color model might describe bistable perception in the #TheDress phenomenon (Uchikawa, Morimoto, & Matsumoto, 2017). The purpose of the present study is to test whether our optimal color hypothesis quantitatively accounts for human observers’ behavior in a wider variety of conditions. Our approach to address this question was to prepare scenes having various shapes of color distributions and to investigate whether observers’ estimations of illuminants followed the prediction by a computational model based on our hypothesis. We conducted three psychophysical experiments designed based on the following two scenarios.

Experiments 1 and 2 used optimal colors for scenes. We then manipulated the degree to which the scenes contained optimal colors. As briefly mentioned above, if a scene contains optimal colors, the optimal color model should function perfectly, as we are able to uniquely find the illuminant under which the optimal color distribution perfectly matches the scene distribution. Therefore, even if the shape of the color distribution changes by darkening some optimal colors in the scene, it is expected that our estimate of illuminant color should not change.

In contrast, we used natural objects in Experiment 3. We implemented a computational optimal color model that incorporated our hypothesis, which predicts an illuminant color from a given color distribution. Guided by this model, we manipulated the shape of the natural color distribution so that the model prediction agreed or disagreed with set illuminant colors (i.e., ground truth). Under this experimental setup, we investigated the degree to which the model predicted observers’ estimation of the illuminant.

In each experiment, we used experimental stimuli that consisted of 61 hexagons spatially packed without a gap, where the surrounding 60 hexagons were designed to have a different shape of color distributions. All experimental stimuli were presented on an experimental monitor, but it was confirmed that they appeared in surface color mode for all observers. The observer's task was to adjust the chromaticity and luminance of the center test hexagon so that it appeared to be a full-white surface under a test illuminant. Results showed that observers’ estimations of illuminant intensity were not well predicted by our model, but other models relying on simple luminance statistics did not predict the observers’ behavior, either. However, estimation of illuminant chromaticity agreed well between human observers and our model prediction. In other words, human observers’ chromaticity settings changed or did not change in a manner predicted by the optimal color model. Although the applicability of our model is limited in some conditions, just as other models are, experimental results support the idea that our visual system can utilize the geometry of the color distribution to estimate the influence of the illuminant.

## General method

### Apparatus

All experiments were computer controlled and conducted in a dark room. Experimental stimuli were presented on a 19-inch cathode-ray tube (CRT) monitor (GDM-520, 1600 × 1200 pixels; Sony Corporation, Tokyo, Japan) controlled by ViSaGe (Cambridge Research Systems, Rochester, UK), which allowed 14-bit intensity resolution for each phosphor. We performed gamma correction using a ColorCAL colorimeter (Cambridge Research Systems) and spectral calibration with a spectroradiometer (PR-650; Photo Research, Inc., Los Angeles, CA). Viewing distance was kept constant by a chin rest positioned 114 cm from the CRT monitor. Observers viewed stimuli binocularly.

### Experimental stimuli

#### Scene geometry

**Figure 3.**
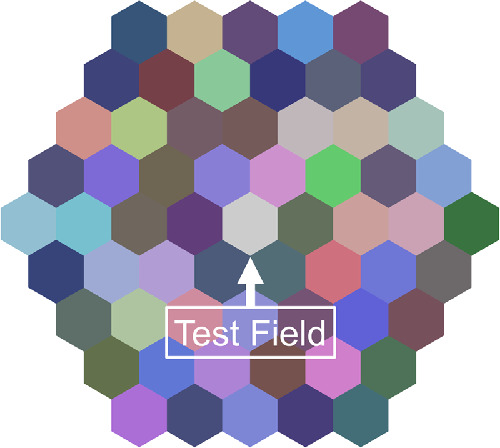
An example of an experimental stimulus configuration that consists of 61 uniformly colored hexagons—the center test field and 60 surrounding stimuli (30 bright colors and 30 dark colors). Each hexagon is subtended 2° diagonally. The chromaticity and luminance of the test field were adjusted by observers. This example is a mountain distribution under 6500 K in Experiment 1. The spatial pattern was shuffled for each trial.

We used 61 hexagons as shown in Figure 3. Each hexagon was 2° diagonally, and the whole stimulus subtended 15.6° width × 14.0° height. The center hexagon was used as a test field, and its chromaticity and luminance were adjustable. The remaining 60 hexagons were surrounding stimuli and were designed to have a specific color distribution, as detailed in each experimental section.

#### Test illuminants

We used 3000 K, 6500 K, and 20,000 K illuminants on the black-body locus as test illuminants for Experiments 1 and 2. For Experiment 3, we used 4000 K, 6500 K, and 10,000 K illuminants.

### Observers

Four observers (KF, KU, MS, and TK; KF, KU, and TK are the co-author of the study) participated in Experiments 1 and 2. Four different observers (HH, HY, RS, and TM; TM is the first author of the study) were recruited for Experiment 3. Observers ages ranged in age between 22 and 63 years (*mean*, 31.5; *SD*, 13.6). All observers had corrected visual acuity and normal color vision as assessed by Ishihara pseudo-isochromatic plates. RS and MS were naïve to the purpose of the study.

### Implementation of optimal color model

Human observers infer the influence of illumination based on the shape of the chromaticity versus luminance distribution in a scene. We implemented this idea based on a computational model that predicts the chromaticity and luminance of a scene illuminant from a set of 60 surface colors. First, we normalized the luminances of the given 60 surfaces so that maximum luminance became 1.0. Thus, the 60 surfaces formed a specific color distribution that peaked at 1.0, and we searched for an optimal color distribution that matched the formed color distribution well. We assumed that the model fully records the optimal color distribution (i.e., the chromaticities and the luminances of any possible optimal color) under 11,457 candidate illuminants: 57 color temperatures from 2000 K to 30,000 K with 500 steps × 201 luminance levels from 1.0 to 3.0 (corresponding to the height of optimal color distribution) with 0.01 steps. Then, the model searches the optimal color distribution that is the best fit for the observed color distribution, assessed by weighted root-mean-square error (WRMSE). If we take a given surface (*S_i_*), its luminance and the luminance of the optimal color at the chromaticity of *S_i_* can be written as *Lsi* and *Loi*, respectively. If we consider all surfaces from *S*_1_ to *S*_60_ in a scene, the WRMSE for a specific candidate illuminant can be calculated by Equation 1:

(1) $$\begin{array}{c} \mathrm{WRMSE}=\sqrt{\frac{\sum_{i=1}^{60}w_{i}{\left(L_{si}-L_{oi}\right)}^{2}}{\sum_{i=1}^{60}w_{i}}}\\ w_{i}=\frac{L_{si}}{L_{oi}} \end{array}$$

We weighted the luminance error by *w_i_* to put a greater weighting on brighter surfaces in proportion to the luminance of the corresponding optimal color. This is based on the finding that brighter colors have a greater influence on an observer's estimation of the illuminant than darker colors (Uchikawa et al., 2012). Under some candidate illuminants, some surface colors might exceed the optimal color distribution, as in the case for illuminants (a), (b), and (d) in Figure 2. Thus, we first need to reject any illuminants under which any of 60 surfaces exceeds the optimal color shell (*Lsi* > *Loi* for any *i*). This restriction allows us to guarantee that we search only illuminants under which all surfaces are physically plausible (i.e., reflectance less than 1.0 at any wavelength). We then look for the illuminant from the remaining candidates that minimizes the value of the WRMSE. The chromaticity and the luminance of the best-fit illuminant give the prediction of the optimal color model. Also note that we restricted our search along the black-body locus in this study because we used test illuminants on the black-body locus, and observers’ settings were also generally found on the locus.

### Procedure

Before the first trial in each experiment, following 1-minute dark adaptation, an observer first adapted for 1 minute to a white screen (2.85 cd/m^2^ equal energy white) covering the whole displayable area of the CRT monitor. Each experiment consisted of nine conditions (three test illuminants × three distributions); thus, nine blocks formed one session. One block had five consecutive repetitions without an intertrial interval. The task of observers was to adjust the chromaticity and the luminance of the test field so that the test surface appeared as a full-white paper placed under a test illuminant. This is the so-called paper-match criterion introduced by Arend and Reeves (1986), and its nature is further argued by Reeves, Amano, and Foster (2008). In a conventional achromatic setting (e.g., Brainard, 1998), only the chromaticity of a test field is adjusted while the luminance is held constant. Our methodology differs in that one response allows us to simultaneously measure the estimated illuminant chromaticity and intensity. Observers used a wired track ball (M570; Logitech, Newark, CA) for 2D adjustment of chromaticity in the MB chromaticity diagram and a number pad for the adjustment of luminance. For each trial, the initial chromaticity was randomly selected from a possible range—L/(L+M), 0.65 ± 0.03; S/(L+M), 1.4 ± 0.33—and the initial luminance was chosen from three possible values (2.86, 5.71, and 14.3 cd/m^2^). There was no time limitation. For each session, one distribution condition was randomly chosen first and fixed. Three illuminant conditions were tested for the distribution condition one after another in a random order. At the beginning of each block, observers adapted to the presented stimulus for 10 seconds (to be briefly adapted to a new illuminant) and then started the adjustment. They re-adapted to the full-white screen for 30 seconds between blocks. For each trial, the spatial arrangement of the 60 surrounding hexagons was shuffled. The observers in total performed two sessions in Experiments 1 and 2 and four sessions in Experiment 3. As a result, we collected 10 white-point settings for each condition in Experiments 1 and 2 and 20 settings in Experiment 3.

## Experiment 1

The purpose of Experiment 1 was to investigate the effect of changing the shape of the chromaticity versus luminance distribution on the estimated illuminant color. We used optimal colors for the surrounding stimuli and manipulated the degree to which a scene contained optimal colors.

### Color distribution of surrounding stimuli

The choice of color combination is important in the framework of the optimal color model. Color distributions for Experiment 1 were designed based on the following considerations. In general, the color distribution of a natural scene with enough reflectance samples typically forms a mountain-like shape (an extreme case is shown in Figure 1). Following this observation, we created a “mountain” condition in which the scene contained a wide range of chromaticities, and their luminance profiles were set to the luminances of optimal colors. Our optimal color models can perfectly predict the ground-truth illuminants for this scene. Then, we lowered the luminances of some colors to generate “reverse” and “flat” shapes, but importantly our model still perfectly predicted the illuminant colors for these two scenes because other optimal colors remained in the scene that allowed our model to predict illuminant colors correctly. The intention behind this manipulation was to determine whether observers’ estimates of illuminant color do not change when the model predictions do not change, even when the apparent shapes of the color distribution change.

**Figure 4.**
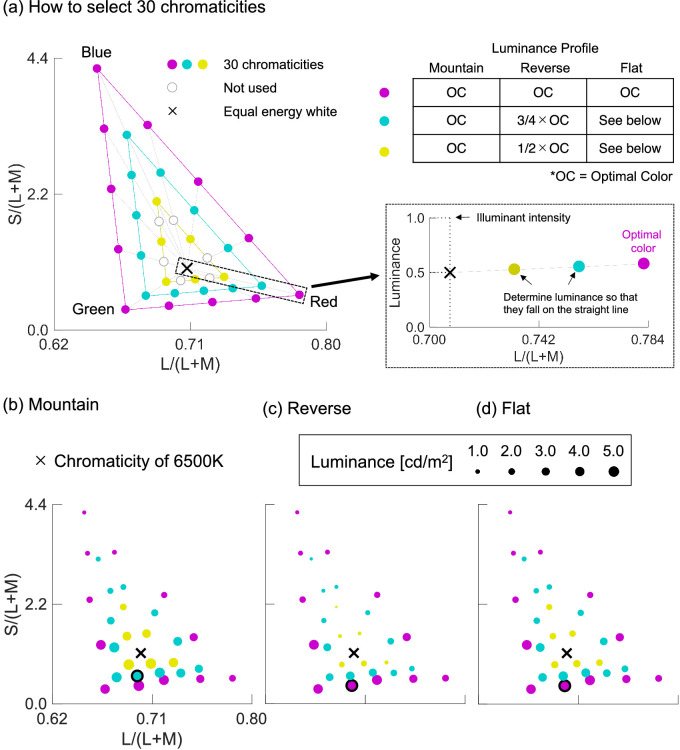
(a) How we selected 30 chromaticities and their luminance values for each distribution condition in Experiment 1; see the main text for details. (b–d) Color distributions for each distribution condition. The symbol size expresses the luminance value at each chromaticity. The highest luminance color is marked by a black edge. The black cross indicates the chromaticity of 6500 K illuminant. Note only the 6500 K condition is shown. Also, only the 30 bright colors are presented here.

Specifically, colors were selected as follows. First, we chose three sets of 60 surfaces (30 bright colors and 30 dark colors) that produce the aforementioned different chromaticity–luminance distributions: mountain, reverse, and flat. The dark colors always had the same chromaticity and 20% of the luminance of the bright colors. Figure 4 helps us to see how 30 chromaticities were chosen and how luminance values were assigned to each chromaticity. We first picked the chromaticities of three colors (labeled Red, Green, and Blue in Figure 4a). The criteria to set these colors were that (a) they did not exceed the chromatic gamut of the CRT monitor under any test illuminant (3000 K, 6500 K, or 20,000 K) and that (b) they had the highest purity possible. We then connected the three colors by straight lines, forming the magenta triangle.

We picked three colors on each side so that the gaps between colors were of equal distance. Next, we drew cyan and yellow triangles that were similar to the magenta triangle (2:3 and 1:3, respectively) and again picked colors on each side in the same way. As shown by the white circles on the yellow triangle, we excluded some colors simply to adjust the number of chromaticities to 30. As a result of this selection, we obtained three purity levels, as shown by the magenta, cyan, and yellow triangles, and each level had 12, 12, and six colors, respectively. Here we note that higher purity colors are more informative regarding the illuminant color according to the framework of the optimal color hypothesis. For example, when a scene had a saturated bright reddish surface, the illuminant was unlikely to be bluish as it could not cover the surface.

Next, we assigned luminance values for each of the 30 chromaticities by defining the surface spectral reflectance for each chromaticity. An inserted table at the top-right corner in Figure 4a shows how luminances were chosen for each distribution condition (mountain, reverse, and flat). The resultant distributions for each distribution condition under 6500 K are shown in Figure 4b, c, and d, where the symbol size indicates the luminance for each chromaticity.

For the mountain distribution, all colors were set to optimal colors. Note that the optimal color distribution peaks at the chromaticity of an illuminant, and the luminance decreases as it gets away from the white point. Thus, for the mountain condition, luminance values were always highest at the yellow symbols and decreased toward the magenta symbols, as shown in Figure 4b. We also note that it is generally not possible to uniquely convert a chromaticity to a surface spectral reflectance simply because there are myriad potential reflectances that could produce the desired chromaticity. However, here we only considered optimal surfaces, allowing us to uniquely find a surface spectral reflectance that produces a desired chromaticity under equal-energy white. In this way, 30 chromaticities were converted to spectral reflectances of optimal colors, with which we simulated the effect of an illuminant change to 3000 K, 6500 K, and 20,000 K.

The reverse and flat conditions were prepared by manipulating the spectral reflectance functions of the mountain distribution. For both the reverse and flat distributions, stimuli with chromaticities on the magenta triangle were again set to optimal surfaces. However, for the reverse distribution, stimuli with chromaticities on the cyan triangle were set to have three-fourths of the reflectance of optimal surfaces at the corresponding cyan chromaticities (i.e., 0.75 and 0.00 reflectance across wavelengths), and stimuli with chromaticities on the yellow triangle were set to have half of the reflectance of optimal surfaces at the corresponding yellow chromaticities (i.e., 0.50 and 0.00 reflectance across wavelengths). As a result, unlike the mountain distribution, luminance values in the reverse condition were highest at the magenta symbols and decreased toward the white point (black cross) as shown in Figure 4c. For the flat distribution, the bottom-right subpanel in Figure 4a helps us to understand our manipulation. The luminance values at the cyan and yellow chromaticities were determined so that they fell on the straight line between the luminance of the optimal color of the magenta triangle and half of the illuminant intensity. As shown in Figure 4d, the resultant distribution appears somewhere between the mountain and reverse conditions, showing relatively flat luminance values over all chromaticities.

We emphasize that chromaticity values for each surface did not change at all depending on the distribution condition; thus, any chromaticity-based illuminant estimation algorithm, such as the mean chromaticity model, predicts exactly the same illuminant color for each distribution. This held for all illuminant conditions. After the selection of surface reflectances, we adjusted the intensity of test illuminants so that mean luminance across the 60 colors became 1.2 cd/m^2^ for all conditions. As a result, illuminant intensities were set to 2.96 cd/m^2^, 2.97 cd/m^2^, and 2.97 cd/m^2^ for mountain, reverse, and flat distributions in the 3000 K condition, respectively. For 6500 K, they were 4.14 cd/m^2^, 4.16 cd/m^2^, and 4.14 cd/m^2^, respectively. Finally, for 20,000 K, we used 3.48 cd/m^2^, 3.49 cd/m^2^, and 3.48 cd/m^2^, respectively. The mean luminance of the surrounding stimuli in Experiment 1 was low. We made the decision to use chromaticities with the highest purity possible and to make sure that participants could find a satisfactory luminance level at which the test field appeared as a white surface (i.e., the upper limit of surface color appearance). Regarding the second point, if the overall light level was too high, the test field might not have appeared white even when the luminance of the test field reached the maximum luminance allowed by our monitor.

### Results and discussion

**Figure 5.**
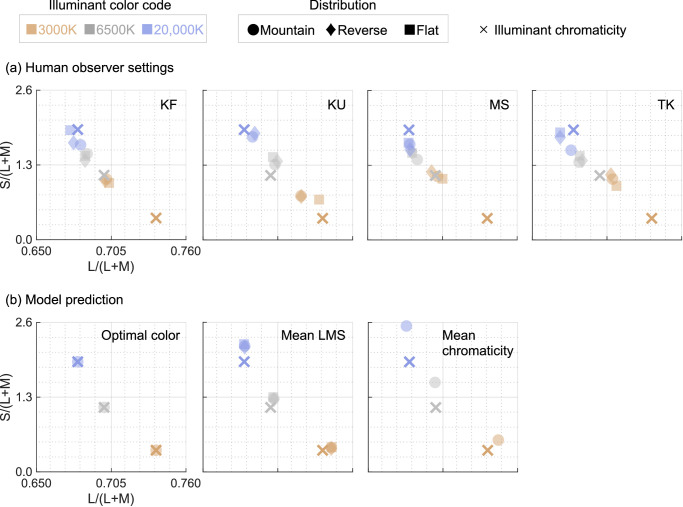
(a) Observer's settings plotted on a MB chromaticity diagram for each condition in Experiment 1. The shape of each symbol indicates distribution condition, and the color indicates the illuminant condition. The colored cross symbols show the chromaticities of the test illuminants. Different subpanels correspond to different observers. (b) Model prediction by the optimal color model, mean LMS model, and mean chromaticity model. Note that the optimal color model and mean chromaticity predict the same chromaticity for any distribution condition by experimental design; thus, only the prediction for the mountain condition is shown for the sake of clarity.

Figure 5a shows the averaged observers’ settings across 10 trials in a MB chromaticity diagram. Error bars are not shown to increase the visibility. The shape of each symbol indicates the distribution condition (mountain, reverse, or flat), and the colors indicate the illuminant conditions (3000 K, 6500 K, or 20,000 K). Different subpanels show the results for different observers. Each colored cross symbol indicates the chromaticity of the test illuminant, thus indicating the ground truth in each illuminant condition.

Figure 5b shows the predictions from the optimal color model, mean LMS model, and mean chromaticity model. Note that in this experiment the optimal color model provided a perfect estimation of illuminant chromaticity regardless of the distribution condition due to the experimental design. For this reason, we plotted only predictions for the mountain condition. This is self-evident, as we used optimal colors for stimuli in this experiment, but we also applied fitting procedures to each of nine conditions based on Equation 1 for a sanity check. This confirmed that predicted chromaticity and intensity for each condition indeed matched the chromaticity and the intensity of a test illuminant.

To calculate the prediction of the mean LMS model, we first averaged cone responses across the 60 surrounding hexagons, and then we converted the averaged cone responses to MB chromaticity coordinates. Note that this manipulation is equivalent to calculating the luminance-weighted average of MB chromaticity coordinates. Here, it can be seen that the chromaticities estimated by mean LMS are positioned very close to the chromaticity of the test illuminants, and there is little difference across distribution conditions under any illuminant condition. The mean chromaticity model provides a prediction based on the average chromaticity across the 60 surrounding hexagons. The predictions are deviated mainly toward a higher S/(L+M) direction from the illuminant chromaticities and do not change depending on the distribution condition. Thus, only predictions for the mountain condition are provided in the figure. In this study, we compared prediction performance between our model and illuminant estimation based on mean chromaticity or mean LMS. This was not intended to rule out more complex color constancy models but rather to evaluate the extent to which simplistic strategies can account for observers’ behaviors.

If an observer is perfectly color constant, the observers’ settings in Figure 5a should superimpose on the chromaticity of the corresponding test illuminant shown by cross symbols; however, that was never the case in this experiment. Instead, all settings showed some deviation from the ground-truth points, and some systematic trends were observed as follows. First, for all observers, we see that the observers’ settings for the three distribution conditions are rather closely clustered. This trend also roughly held for any illuminant condition, suggesting that the shape of the chromaticity–luminance distribution had little impact on the observers’ estimation of the illuminant chromaticity in this experiment, as predicted by the optimal color model, mean LMS model, and mean chromaticity model as shown in Figure 5b.

Second, as predicted, the observers’ settings were separated depending on the illuminant condition. Thus, unsurprisingly, the color temperature of the test illuminant had a strong effect on the observers’ settings. The degree of separation across the illuminant condition appears to depend on the observers; for example, settings for observer MS were closely gathered across test illuminants whereas those for observer KU showed a greater separation.

**Figure 6.**
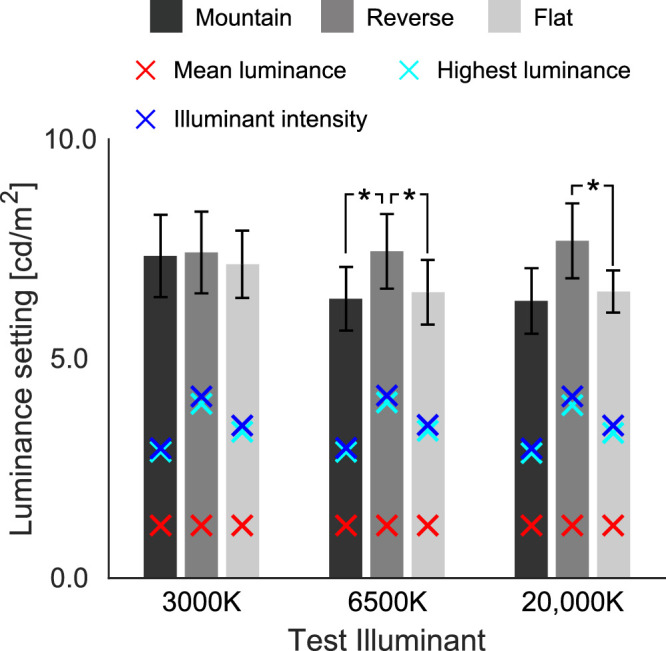
Observer's luminance settings for each condition in Experiment 1. The distribution condition is labeled by the lightness of the bars. The red cross symbols indicate the mean luminance across the 60 surrounding surfaces, and the cyan symbol shows the highest luminance of the 60 surfaces. The blue cross symbols show the intensity of the set test illuminant and therefore indicate the prediction by the optimal color model. The error bars indicate ±*SE* across four observers. Asterisks show a significant difference (α < 0.05, Bonferroni's correction).

Our methodology allowed observers to simultaneously adjust luminance in addition to chromaticity, which provided an indication of each observer's estimation of illuminant intensity. Figure 6 shows the luminance setting for each condition, averaged across four observers. To determine whether the observers’ settings showed agreement with simple luminance statistics, red and cyan crosses indicate the mean luminance and the highest luminance across the 60 surrounding surfaces, respectively. Blue crosses show the intensities of test illuminants (i.e., ground truth); thus, if an observer's setting matches the blue crosses, that indicates that the observer perfectly estimated the intensity of the illuminant. Note that in this experiment the optimal color model provided the perfect estimation of illuminant intensity for all conditions by design, so the blue cross symbols also indicate the prediction of the optimal color model. The observers’ estimations of intensity were never perfect, however. Instead, their settings generally exceeded the set illuminant intensity, showing that the observers assumed the illuminants were much more intense than they actually were.

To quantitatively evaluate whether the pattern of luminance settings can be explained by simple luminance statistics, we calculated correlation coefficient between the observers’ average settings and each luminance statistic across nine conditions. Correlation coefficients were 0.0788 (*p* > 0.05), 0.710 (*p* = 0.0320), and 0.700 (*p* = 0.0359) for mean luminance, highest luminance, and illuminant intensity, respectively. Thus, the highest luminance model and illuminant intensity (i.e., prediction from the optimal color model) showed a significant correlation. It is possible that observers used different statistics or a more complicated strategy, but the findings suggest that their behaviors can be explained reasonably well by the optimal color model or simple luminance statistics (highest luminance in this case).

Next, a two-way, repeated-measures analysis of variance (ANOVA) was performed with distribution condition (mountain, reverse, or flat) and illuminant condition (3000 K, 6500 K, or 20,000 K) as the within-subject factors for the luminance settings. The main effects of distribution condition and illuminant condition were not significant: *F*(2, 6) = 3.48, *p* > 0.05; *F*(2, 6) = 3.88, *p* > 0.05, respectively. However, the interaction between the two factors was significant: *F*(4, 12) = 5.45, *p* = 0.00975. Further analysis of the interaction revealed that the simple main effect of distribution condition was significant at 6500 K and 20,000 K, *F*(2, 6) = 9.71, *p* = 0.0132; *F*(2, 6) = 7.80, *p* = 0.0214, respectively, but not at 3000 K, *F*(2, 6) = 0.15, *p* > 0.05. Furthermore, the simple main effect of illuminant condition was significant for the mountain condition, *F*(2, 6) = 5.48, *p* = 0.0443, but was not significant for the reverse and flat conditions, *F*(2, 6) = 1.39, *p* > 0.05; *F*(2, 6) = 3.76, *p* > 0.05, respectively.

To further clarify the relation across levels regarding the simple main effects of distribution and illuminant, the results of multiple comparisons with Bonferroni's correction (significance level α = 0.05) where significant differences were found are indicated by asterisks in Figure 6. Overall, the results of these statistical analyses suggest that the luminance settings were higher for the reverse condition than for the mountain and flat conditions at 6500 K, and they were higher for the reverse condition than for the flat condition at 20,000 K.

Next, to examine the extent to which color constancy held for each condition, it would be helpful to quantify the degree of constancy. Several metrices have been used in past studies (summarized in Foster, 2011), but a fundamental idea is to capture how much the observers’ subjective white points shifted in relation to physical changes of illuminant chromaticity. In this study, we used an index that considers both the direction and the amount of the observers’ settings shift.

**Figure 7.**
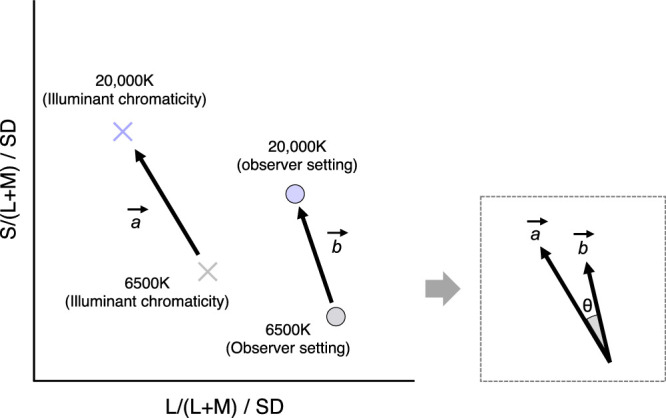
How to define the CI. Vectors $\vec{a}$ and $\vec{b}$ show the shift in physical illuminant chromaticity and the shift in the perceptual white point, respectively, and θ is the angle between the two vectors. Note that each axis was divided by the average of standard deviations across four observers for the mountain 6500 K condition to compensate for the scale difference along L/(L+M) and S/(L+M). The CI is defined by $|\vec{b}\left|cos\theta /\right|\vec{a}|$ in this scaled MB chromaticity diagram.

As shown in Figure 7, we first defined two vectors, $\vec{a}$ and $\vec{b}$, where $\vec{a}$ is a vector originating from the chromaticity of the 6500 K illuminant to the illuminant chromaticity of 3000 K or 20,000 K. The vector $\vec{b}$ extends from the observers’ settings under 6500 K to settings under the test illuminant (3000 K or 20,000 K). We defined θ as indicating the angle that two vectors create, as shown in right panel in Figure 7. The constancy index (CI) is calculated by Equation 2:

(2) $$\mathrm{CI}=\;\left|\vec{b}\right|c\mathrm{os}\theta /\left|\vec{a}\right|$$

If the shift of an observer's setting caused by illuminant change is the same as the shift of illuminant chromaticity in terms of both distance and direction, then the CI becomes 1.0. Any deviation in distance or direction would lower the CI. In theory, the CI can take a value more than 1.0, but we did not find such a case in this study. If the direction of shift perfectly matches between $\vec{a}$ and $\vec{b}$ (i.e., θ = 0), then cosθ becomes 1.0 and thus does not lower the CI value. In this sense, cosθ serves as a loss factor due to the directional deviation.

Also note that a problem with calculating a distance-based metric in an MB chromaticity diagram is that scales for L/(L+M) and S/(L+M) are different and arbitrary. Thus, to give approximately equal consideration to both directions, we divided each axis by the average of standard deviations across four observers of the settings under the mountain 6500 K condition (which was considered to be the standard condition). All CIs were calculated in this scaled MB chromaticity diagram. Our metric is not the only way to quantify color constancy, but we confirmed that other common constancy indices agreed well with our implementation (see Supplementary Material).

**Figure 8.**
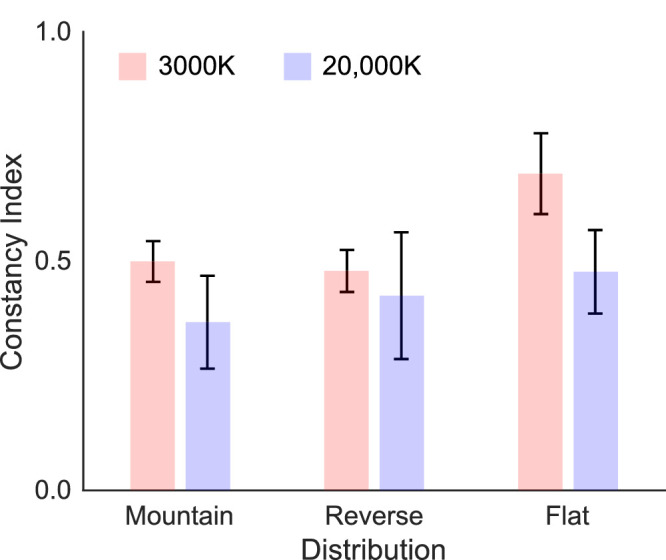
The CI averaged across four observers in Experiment 1. Note that the CIs closer to 1.0 indicate better color constancy. Error bars indicate ±*SE* across four observers.

The bar chart in Figure 8 shows averaged CIs across four observers for each condition. First it was shown that the CIs were roughly around 0.5 for the mountain and reverse conditions at 3000 K, and the CIs were slightly lower for 20,000 K. The flat condition had a slightly higher CI, especially for 3000 K.

We performed two-way, repeated-measures ANOVAs with distribution condition (mountain, reverse, or flat) and illuminant condition (3000 K or 20,000 K) as the within-subject factors for the CIs. The main effect of distribution condition was significant, *F*(2, 6) = 8.40, *p* = 0.0182, whereas the main effect of the illuminant conditions and the interaction between two factors were not significant, *F*(1, 3) = 7.63, *p* > 0.05; *F*(2, 6) = 0.59, *p* > 0.05, respectively.

Multiple comparisons with Bonferroni's correction (significance level α = 0.05) for the distribution condition found that the flat condition showed higher CIs than the mountain and reverse conditions, but there was no significant difference between the mountain and reverse conditions.

Overall, the statistical analyses indicated that color constancy worked equally well under 3000 K and 20,000 K, and it also worked better for the flat distribution than for the other distributions in Experiment 1. We note that the use of a low luminance level for the surrounding stimuli might have introduced rod intrusion on the color appearance of the surrounding stimuli and test field. However, the CI is a relative measure and thus partially suppresses the influence of rod intrusion.

**Figure 9.**
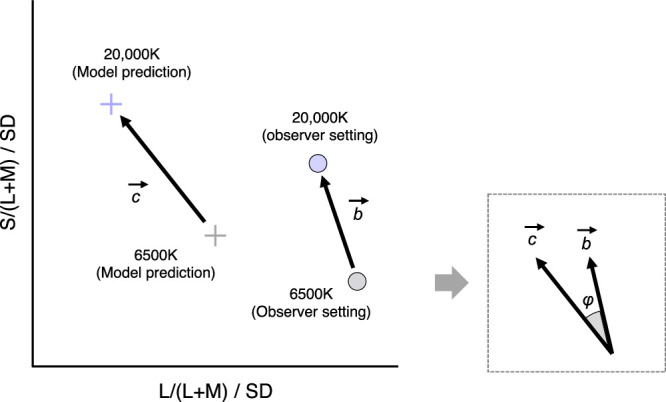
How to define the MI that quantifies the degree to which the three models of interest (optimal color, mean LMS, and mean chromaticity) account for the human observers’ settings. Vectors $\vec{c}$ and $\vec{b}$ show the shift of chromaticities predicted by a model and the shift of the perceptual white point, respectively. φ is the angle between two vectors. MI is defined by $|\vec{b}\left|cos\varphi /\right|\vec{c}|$ in the scaled MB chromaticity diagram.

We have so far discussed the degree to which human observers achieved color constancy; however, the main purpose of the present study was to quantify how well our optimal color models accounts for the pattern of human observers’ settings, ideally in comparison with other candidate color constancy models. The CI calculates how much an observer's setting shifted in relation to a shift of the physical illuminant chromaticity. Thus, it becomes higher when shifts in the perceptual white point and physical illuminant are close to each other in distance and direction, and it becomes 1.0 when the two shifts perfectly match. Although the CI was originally designed to reflect the degree of color constancy, we can also consider a metric that indicates the degree to which the shift of illuminant chromaticities predicted from computational models agrees with that of the observers’ white-point settings. Based on this idea, we quantified the degree to which our optimal color model and other computational models (mean LMS and mean chromaticity) predicted human observers’ settings. We introduced the model index (MI) as defined by Equation 3, which replaces the × symbols in Figure 7 with + symbols, indicating predictions from a model as shown in Figure 9:

(3) $$\begin{array}{c} \mathrm{MI}=\left|\vec{b}\right|\cos\varphi /\left|\vec{c}\right|\; \end{array}$$

**Figure 10.**
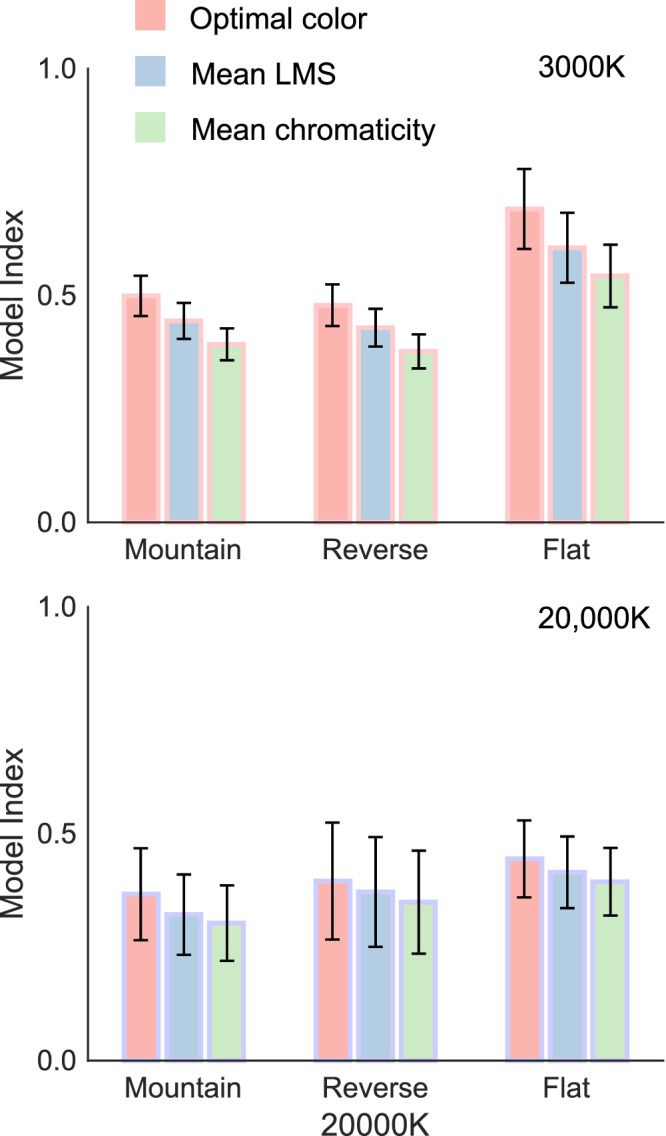
The MI for each condition in Experiment 1. The MIs were calculated separately for each observer first and then averaged across four observers. A higher MI indicates that a model predicted the human observers’ settings better. Error bars show ±*SE* across four observers.

Higher values indicate better model prediction, and 1.0 indicates perfect prediction. Figure 10 shows the MIs in each condition. Note that in this experiment the optimal color model predicted illuminant chromaticity perfectly; therefore, the MI values match those of the CIs. The mean LMS model first averaged cone signals across 60 surfaces, and the average cone signal was transformed to MB chromaticity coordinates. The mean chromaticity estimates illuminant color based on the average chromaticity across 60 surfaces.

We can see that the MI values are high in the order of optimal color, mean LMS, and mean chromaticity models for all conditions. We ran the following analysis to confirm if our model prediction was statistically better than that of the mean LMS and mean chromaticity models. We performed a three-way, repeated-measures ANOVAs with model type (optimal color, mean LMS, and mean chromaticity), distribution condition (mountain, reverse, and flat), and illuminant condition (3000 K and 20,000 K) as the within-subject factors for the MIs. The main effects of the model type and distribution condition were significant, *F*(2, 6) = 52.8, *p* = 0.000155; *F*(2, 6) = 7.93, *p* = 0.0207, respectively, whereas the main effect of illuminant condition was not significant, *F*(1, 3) = 8.51, *p* > 0.05. The interactions between model type and distribution condition and between model type and illuminant condition were significant, *F*(4, 12) = 15.4, *p* = 0.000113; *F*(2, 6) = 220.9, *p* < 0.00001, respectively. In contrast, the interaction between distribution condition and illuminant condition was not significant, *F*(2, 6) = 0.57, *p* > 0.05. The interaction among three factors also was not significant, *F*(4, 12) = 3.11, *p* > 0.05.

We then analyzed simple main effects for the interaction between model type and distribution condition. The simple main effect of model type was significant for all distribution conditions: *F*(2, 18) = 45.4, *p* < 0.00001 for the mountain condition; *F*(2, 18) = 42.9, *p* < 0.00001 for the reverse condition; and *F*(2, 18) = 62.8, *p* < 0.00001 for the flat condition. Also, the simple main effect of the distribution condition was significant for all models: *F*(2, 18) = 8.36, *p* = 0.00271; *F*(2, 18) = 7.74, *p* = 0.00375; and *F*(2, 18) = 7.69, *p* = 0.00386, respectively. Post hoc multiple comparison using a Bonferroni's correction (significance level, 0.05) revealed the following: (a) mountain = reverse, mountain < flat, and reverse < flat for the optimal color model; and (b) mountain = reverse, mountain < flat, and reverse = flat for the mean LMS and mean chromaticity models. We also found the following results: optimal color model > mean LMS model, optimal color model > mean chromaticity model, and mean LMS model > mean chromaticity model for all distribution conditions (i.e., mountain, reverse, and flat distributions).

Next, we analyzed simple main effects of interaction between model type and illuminant condition. We found that model type showed significant simple main effects at both 3000 K and 20,000 K, *F*(2, 12) = 97.7, *p* < 0.00001; *F*(2, 12) = 19.8, *p* = 0.000158, respectively. Moreover, the simple main effects of illuminant condition were significant for the optimal color model and mean LMS model, *F*(1, 9) = 12.5, *p* = 0.00636; *F*(1, 9) = 8.78, *p* > 0.0159, respectively, but were not significant for the mean chromaticity model, *F*(1, 9) = 4.81, *p* > 0.05. Again multiple comparison using a Bonferroni's correction (significance level, 0.05) provided the following results: (a) optimal color model > mean LMS model, optimal color model > mean chromaticity model, and mean LMS model > mean chromaticity model for 3000 K; and (b) optimal color model > mean LMS model, optimal color model > mean chromaticity model, and mean LMS model = mean chromaticity model for 20,000 K. Overall, these statistical tests suggest that our optimal color model predicted observers’ settings generally better than the mean LMS and mean chromaticity models, at least under the current experimental procedure. This observation held for the different distributions and test illuminants, as supported by the results from multiple comparisons reported above.

With regard to the degree of color constancy, it was shown that human observers can achieve roughly 50% constancy for each distribution condition, although the flat condition showed slightly higher CIs. MI-based analysis revealed that our optimal color model predicted the shift of observers’ settings in response to test illuminant changes better than other candidate models in general. With regard to raw chromaticity settings (Figure 5a), our model was successful in revealing that the shape of distribution had little impact, but the mean LMS model and mean chromaticity model also predicted this observation (Figure 5b). In Experiment 2, to further pursue the applicability of our model, we tested three different distributions designed to separate predictions made by our model and the mean LMS model. We designed the shape of color distributions so that optimal color model would again perfectly predict the chromaticity of the illuminant, but the mean LMS model predicted a different illuminant for each distribution condition. Our intent was to determine the effect of the shape of the color distribution on human observers’ white-point settings, as predicted by the optimal color model.

## Experiment 2

Experiment 2 tested follow-up conditions from Experiment 1, and color distribution shapes were designed based on the mountain condition in Experiment 1. The luminance profiles of the mountain condition were modulated to alter the prediction from the mean LMS model while keeping the prediction of optimal color model constant.

### Color distribution of surrounding stimuli

**Figure 11.**
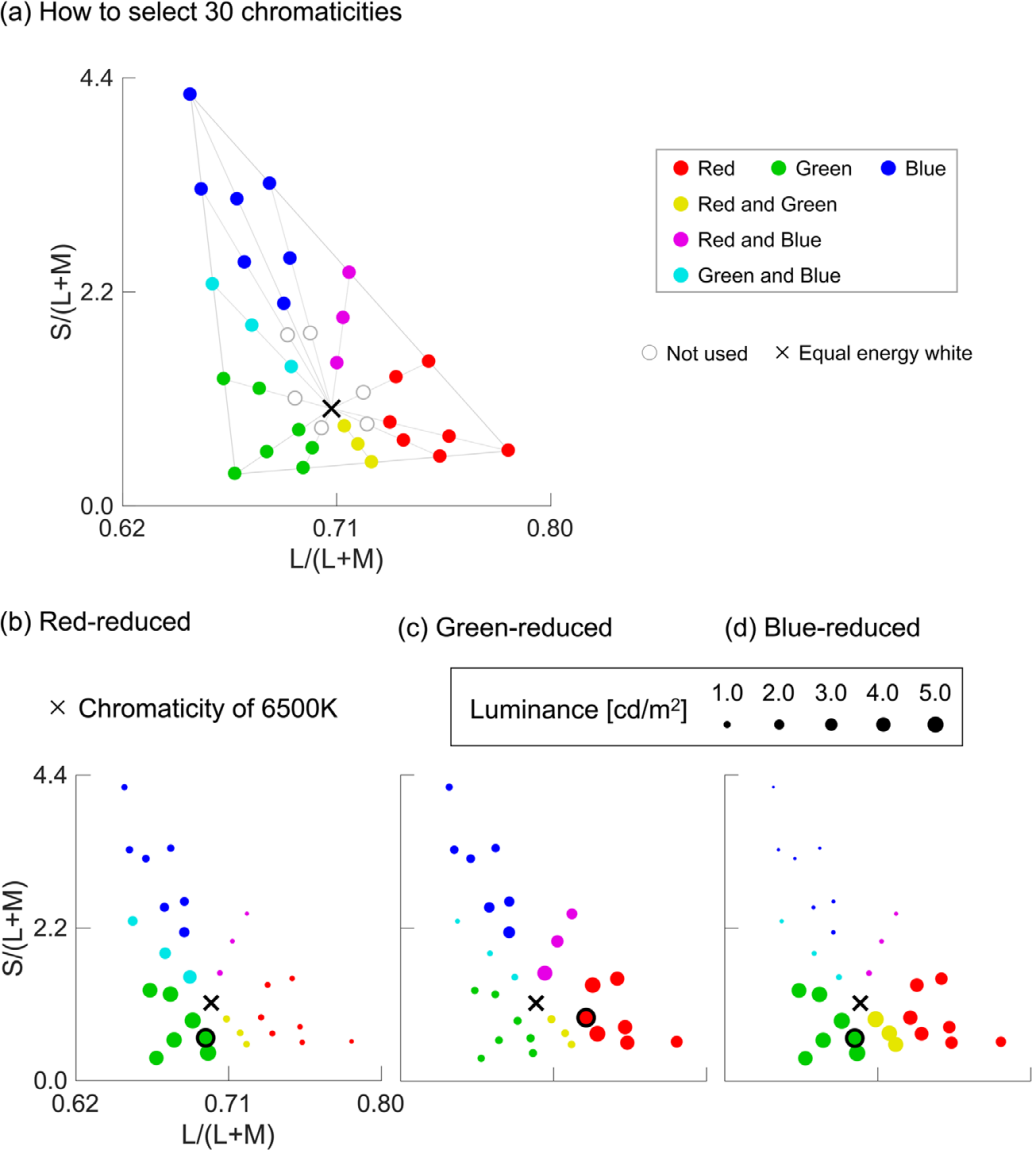
(a) How to group 30 chromaticities into six different categories. Note that the yellow, magenta, and cyan symbols indicate colors that belonged to both red and green, both red and blue, and both green and blue groups, respectively. (b–d) Color distributions for each distribution under 6500 K. The size of the symbols indicates the luminance value at each chromaticity. The highest luminance color is marked by a black edge. Only the 30 bright colors are shown here.

We used three new distributions: red-reduced, green-reduced, and blue-reduced. We used the same 30 chromaticities as in Experiment 1, but luminance values were assigned differently as follows. First, as shown in Figure 11a, we divided the 30 chromaticities into six categories. Some chromaticities were included in only a red, green, or blue group (indicated by red, green, and blue circles, respectively). But, some chromaticities belonged to more than one group (e.g., magenta circles belong to both red and blue groups). For the red-reduced distribution, colors included in the red group (i.e., red, magenta, and yellow symbols) were set to have one-third the luminance of optimal colors, whereas other colors were all set to optimal colors. The same manipulation was applied to the green-reduced and blue-reduced conditions. The intent of this manipulation was to reduce the contribution to mean LMS values from colors in a specific region in the chromaticity diagram. For example, for the green-reduced distribution, colors that have low L/(L+M) values are forced to have low luminance, and thus the mean LMS values across 60 surfaces should be biased towards higher L/(L+M). We used 30 bright colors and 30 dark colors that had the same chromaticity but 20% of the luminance of the bright colors. The luminance values over chromaticities under the test illuminant of 6500 K are shown in Figure 11b to d. Note that for each distribution some colors had noticeably lower luminances than others.

In Experiment 2 we also set the intensity of the test illuminants such that the mean luminance across the 60 colors became 1.2 cd/m^2^. To achieve this, illuminant intensities were set to 4.45 cd/m^2^, 4.29 cd/m^2^, and 4.18 cd/m^2^ for red-reduced, green-reduced, and blue-reduced distributions in the 3000 K condition. For 6500 K, they were 4.53 cd/m^2^, 4.63 cd/m^2^, and 4.63 cd/m^2^, respectively; for 20,000 K, we used 3.66 cd/m^2^, 3.74 cd/m^2^, and 3.80 cd/m^2^, respectively.

### Results and discussion

**Figure 12.**
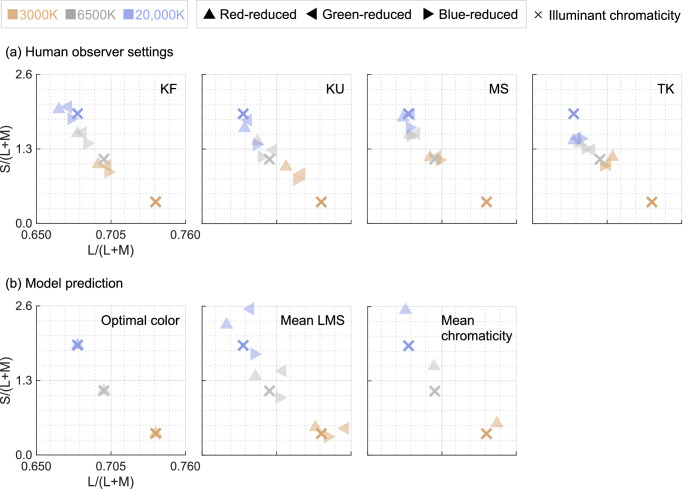
(a) The observers’ white settings for each condition in Experiment 2. Different panels indicate different observers. (b) Model prediction by the optimal color model, mean LMS model, and mean chromaticity model. The optimal color model and mean chromaticity model predict the same chromaticity regardless of distribution condition by experimental design; thus, only the prediction for the red-reduced condition is shown here.

The presentation of results follows the format in Experiment 1. Figure 12a shows the averaged observers’ settings across 10 trials. The shape of the symbol indicates the distribution condition (red-reduced, green-reduced, or blue-reduced), and the colors indicate the illuminant condition (3000 K, 6500 K, and 20,000 K). Figure 12b shows predictions from the optimal color model, mean LMS model, and mean chromaticity model. As in Experiment 1, the optimal color model provided perfect estimation of illumination for all conditions by design; thus, we only plotted predictions for the red-reduced condition. Predictions from the mean LMS model show some differences depending on distribution condition as expected. For example, for the red-reduced condition, surfaces with high L/(L+M) had lower luminance than the other surfaces, thus contributing less to the averaged LMS values. As a result, the prediction shifted toward the lower L/(L+M) direction. The mean chromaticity model predicted exactly the same chromaticity as Experiment 1 because our experimental manipulation changed only the luminance profiles of the surrounding stimuli while keeping the chromaticities of the 60 surfaces constant.

For observer MS and TK, we see that their settings for the three distribution conditions seem to come close under any illuminant condition, which is consistent with the prediction from the optimal color model. However, some separation and systematic pattern of settings were observed for KU and especially KF—lower L/(L+M) for the red-reduced condition, higher L/(L+M) for the green-reduced condition, and lower S/(L+M) for the blue-reduced condition—observations that seem to be somewhat consistent with the predicted pattern by the mean LMS model. Also, the observers’ settings were predictably influenced by the color temperature of the illuminant.

**Figure 13.**
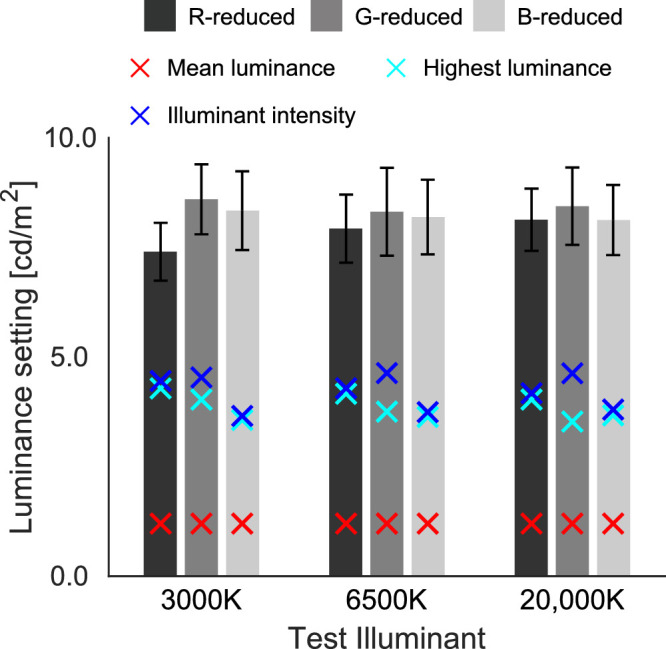
The observers’ luminance settings for each condition in Experiment 2. Distribution conditions are indicated by the lightness of the bars. The red and cyan crosses indicate the mean luminance and the highest luminance across 60 surrounding surfaces, respectively. The blue cross symbol shows the intensity of the test illuminant. Error bars are ±*SE* across four observers.

Figure 13 shows the averaged luminance settings across four observers for each condition. The predictions for mean luminance, highest luminance, and illuminant intensity are indicated by red, cyan, and blue cross symbols, respectively. We performed two-way, repeated-measures ANOVAs with the distribution condition (red-reduced, green-reduced, or blue-reduced) and illuminant condition (3000 K, 6500 K, or 20,000 K) as the within-subject factors for the luminance settings. The main effect of distribution condition and illuminant condition were not significant: *F*(2, 6) = 0.10, *p* > 0.05; *F*(2, 6) = 1.65, *p* > 0.05, respectively. The interaction between two factors also was not significant, *F*(4, 12) = 0.55, *p* > 0.05. Thus, there was no significant difference for any pair of conditions in Experiment 2.

We again calculated correlation coefficients between the nine observers’ settings and each luminance statistics. The correlation coefficients were –0.356 (*p* > 0.05), –0.6273 (*p* > 0.05), and 0.0210 (*p* > 0.05) for mean luminance, highest luminance, and illuminant intensity (i.e., prediction from the optimal color model), respectively. Thus, these models failed to predict the human observers’ performance. This might be due to the observers’ use of a more complex strategy than the simple luminance statistics tested here.

**Figure 14.**
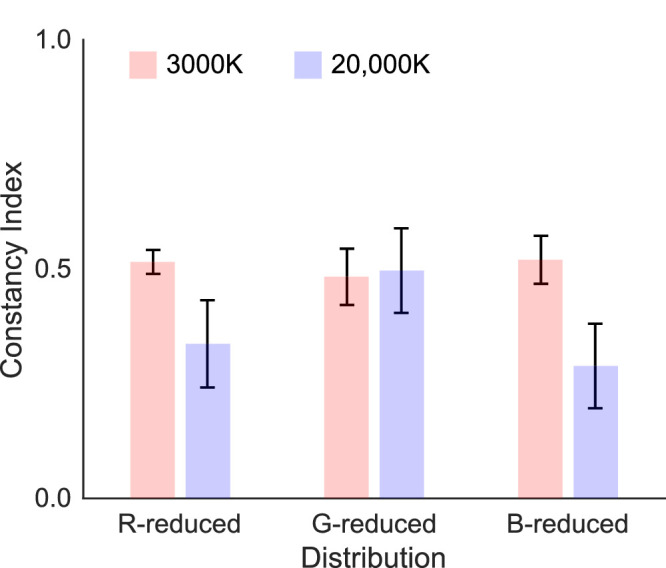
The CI for each condition in Experiment 2. CIs closer to 1.0 indicate a higher degree of color constancy. Error bars show ±*SE* across four observers.

Next, we quantified the degree of color constancy based on Equation 2. Figure 14 shows the CIs for all conditions. We conducted two-way ANOVAs with the distribution condition (red-reduced, green-reduced, or blue-reduced) and illuminant condition (3000 K or 20,000 K) as the within-subject factors for the CIs. The main effect of distribution condition was not significant: *F*(2, 6) = 3.59, *p* > 0.05. Also, the main effect of illuminant condition and the interaction between two factors were not significant: *F*(1, 3) = 7.18, *p* > 0.05; *F*(2, 6) = 2.16, *p* > 0.05, respectively. Consequently, the degree of color constancy was not influenced by the shape of the distribution or the color temperature of the test illuminants.

**Figure 15.**
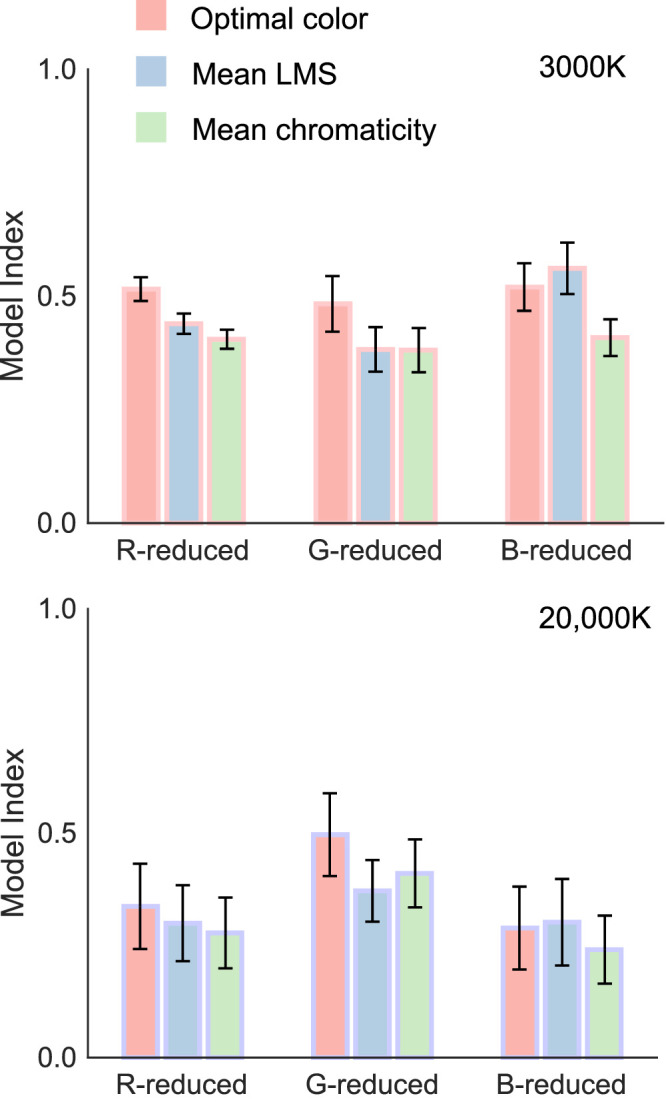
The MI for each condition in Experiment 2. Higher values indicate better prediction by the models. Error bars show ±*SE* across four observers.

We again calculated MIs from Equation 3 based on predictions from the optimal color, mean LMS, and mean chromaticity models as shown in Figure 15. For the 3000 K condition (top nine bars), it was found that the MI values were generally higher for the optimal color model than for the other models, except for the blue-reduced condition, where the mean LMS model showed a slightly higher MI than that of the optimal color model. This observation also held for the 20,000 K condition. However, the error bars are large, thus making it difficult to make a conclusive statement about whether our optimal color model overall worked better than other models. For this reason, we performed the following statistical analyses.

Three-way, repeated-measures ANOVAs were conducted with model type (optimal color, mean LMS, or mean chromaticity), distribution condition (red-reduced, green-reduced, or blue-reduced), and illuminant condition (3000 K or 20,000 K) as the within-subject factors for the MIs. The main effect of model type was significant, *F*(2, 6) = 68.1, *p* = 0.000075, whereas the main effects of distribution condition and illuminant condition were not significant: *F*(2, 6) = 1.27, *p* > 0.05; *F*(1, 3) = 6.82, *p* > 0.05, respectively. The interaction between model type and distribution condition and between model type and illuminant condition were significant: *F*(4, 12) = 48.1, *p* < 0.00001; *F*(2, 6) = 18.6, *p* < 0.00268, respectively. In contrast, the interaction between distribution condition and illuminant condition was not significant, *F*(2, 6) = 2.18, *p* > 0.05. The interaction among three factors also was not significant, *F*(4,12) = 3.02, *p* > 0.05.

Regarding the significant interaction between model type and distribution condition, the simple main effect of model type was significant under the red-reduced, green-reduced, and blue-reduced conditions: *F*(2, 18) = 38.7, *p* < 0.00001; *F*(2, 18) = 75.6, *p* < 0.00001; *F*(2, 18) = 64.0, *p* < 0.00001, respectively. In addition, the simple main effect of distribution condition was significant for the optimal color model, *F*(2, 18) = 4.79, *p* = 0.0184, but was not significant for the mean LMS model or mean chromaticity model: *F*(2, 18) = 2.79, *p* > 0.05; *F*(2, 18) = 3.36, *p* > 0.05, respectively. Furthermore, multiple comparison using a Bonferroni's correction (significance level, 0.05) showed that red-reduced = green-reduced; red-reduced = blue-reduced; and green-reduced > blue-reduced for the optimal color model. Also, optimal color > mean LMS, optimal color > mean chromaticity, and mean LMS > mean chromaticity for both the red-reduced and blue-reduced conditions. For the green-reduced condition, optimal color > mean LMS model, optimal color > mean chromaticity, and mean LMS model = mean chromaticity.

Next, we analyzed the simple main effects of interactions between model type and illuminant condition. We found that model type showed significant simple main effects at 3000 K and 20,000 K: *F*(2, 12) = 79.7, *p* < 0.00001; *F*(2, 6) = 31.0, *p* = 0.000018, respectively. Moreover, the simple main effects of illuminant condition were significant for the optimal color model and mean LMS model, *F*(1, 9) = 8.27, *p* = 0.0183; *F*(1, 9) = 8.88, *p* = 0.0155, respectively, but they were not significant for the mean chromaticity model, *F*(1, 9) = 3.73, *p* > 0.05. Again, multiple comparison using a Bonferroni's correction (significance level, 0.05) showed that optimal color > mean LMS, optimal color > mean chromaticity, and mean LMS > mean chromaticity at 3000 K. At 20,000 K, optimal color > mean LMS, optimal color > mean chromaticity, and mean LMS = mean chromaticity.

Our main interest in this experiment was to evaluate whether our proposed model preforms better than other candidate models. The calculated MIs and statistical analyses overall support this idea, which is consistent with the trends in Experiment 1. However, predictions from the optimal color model are still limited in Experiments 1 and 2, as MIs for the optimal color model were considerably smaller than 1.0, showing a lack of agreement. Thus, it is still inconclusive as to whether the optimal color model is a good candidate strategy to be adopted by human observers to infer the influence of illuminant.

It is worth noting that we used optimal surfaces for the surrounding stimuli that do not exist in the real world; thus, the observers never experienced luminance distributions formed by optimal colors. Instead, in the real world, the visual system encounters scenes containing natural objects that have a reflectance less than 1.0. In Experiment 3, we used 60 reflectances selected from a database of 575 natural objects (Brown, 2003). We manipulated the shape of the distribution to “deceive” the optimal color model, so that even when the test illuminant was held constant the model predicted different illuminant chromaticities influenced by the shape of the color distribution. We then measured the observers’ white points using the same procedure as in Experiments 1 and 2. We tested whether the white points of human observers change in response to a change in the shape of the color distribution, and, if so, whether the optimal color model can predict those shift patterns.

## Experiment 3

### Color distribution of surrounding stimuli

We used three distributions: natural, red-increased, and blue-increased. As discussed in the previous section, the aim of manipulating color distributions in this experiment was to alter the prediction by the optimal color model. To achieve this, we selected 60 reflectances from a database that contains 575 spectral reflectances of natural objects (Brown, 2003) based on the following criteria. First, a white surface provides a direct cue to the illuminant color, thus allowing observers to adjust the color of the test field so that it simply matches the white surface. Therefore, we decided to exclude 59 flat reflectances that have chromaticities in a range between 0.6978 and 0.7178 along the L/(L+M) axis and between 0.800 and 1.200 along the S/(L+M) axis when rendered under equal-energy white. Second, we randomly sampled 60 surfaces from the remaining 516 reflectances so that mean chromaticity across the 60 surfaces corresponded to the chromaticity of equal-energy white—0.7078 for L/(L+M) and 1.0000 and S/(L+M)—when rendered under equal energy to make a scene chromatically balanced. The chosen 60 surfaces were defined as the natural distribution. This natural distribution condition was designed to form a mountain-like color distribution to imitate the scene distribution typically seen in the natural environment. We also made sure that the optimal color model was able to perfectly estimate the chromaticity of the test illuminant in this condition.

Next we manipulated the luminance profile of natural distribution to alter the model prediction without changing the chromaticity of any surface. To create the red-increased condition, we scaled up or down 60 reflectances by multiplying scalar values without changing the spectral shape of the reflectances. The scalar value was determined proportionally to the L/(L+M) of the reflectance under equal-energy white so that reflectances with higher L/(L+M) had higher luminance. Similarly, to create the blue-increased distribution, we scaled 60 reflectances by the scalar value as a function of S/(L+M) so that the reflectances with high S/(L+M) became lighter. We made sure that maximum reflectance did not exceed 1.0 to keep all reflectances physically plausible.

**Figure 16.**
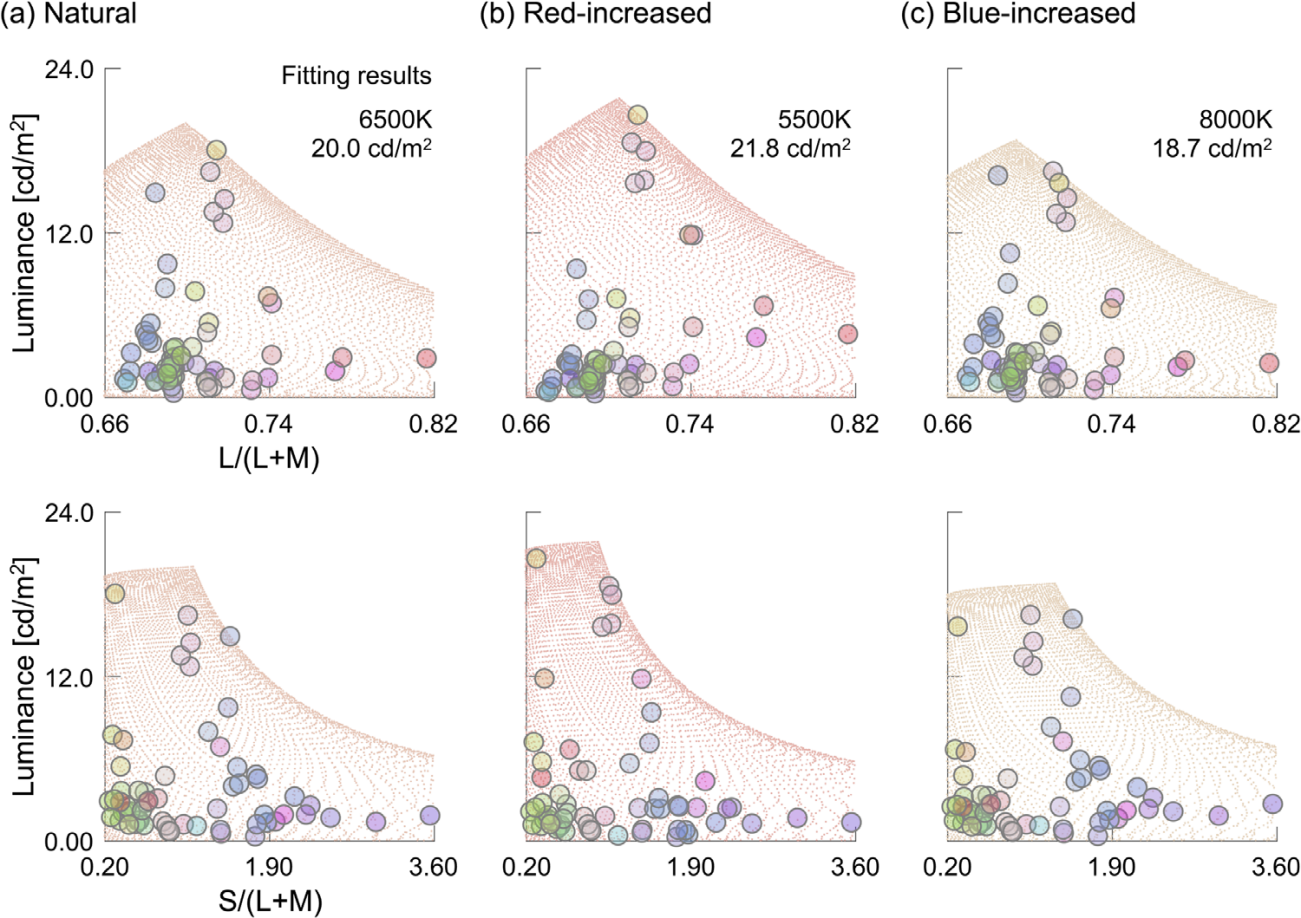
Color distributions for each distribution condition under 6500 K in Experiment 3. Each panel also indicates the optimal color distribution best fit to the 60 colors.

Resultant distributions are shown in Figure 16. We see that the natural distribution has a mountain-like shape, whereas the red-increased and blue-increased conditions both have skewed luminance profiles. For the red-increased distribution, the upper subpanel shows that luminances around higher L/(L+M) regions are elevated. Also, for the blue-increased distribution, the lower subpanel shows that luminances around higher S/(L+M) regions are increased. Importantly, fitted optimal color distributions are plotted together, and best-fit color temperature and illuminant intensity are shown at the top right of each upper subpanel. These fitting results indicate that model perfectly predicted the color temperature for the natural condition. In contrast, the estimation is biased at 5500 K and 8000 K for the red-increased and blue-increased distributions, respectively, as expected. For the 4000 K condition, the model predictions of color temperature were 4000 K, 3500 K, and 4500 K for the natural, red-increased, and blue-increased distributions, respectively. For the 10,000 K condition, the predictions were 10,000 K, 7500 K, and 15,500 K, respectively.

Note that different color temperatures were used for the test illuminants in Experiment 3 (4000 K, 6500 K, and 10,000 K) compared to Experiments 1 and 2 (3000 K, 6500 K, and 20,000 K). This is because we wanted to manipulate distribution shapes so that the model predicted higher or lower color temperatures than the ground truth. If we had instead used 20,000 K as a test illuminant, for example, the model would have predicted a color temperature of 30,000 K for the blue-increased distribution, and the observers’ chromaticity settings might have exceeded the chromatic gamut of the CRT display. For this reason, we employed less extreme color temperatures in this experiment.

We set intensities of test illuminants so that the mean luminance across 60 surfaces became 6.0 cd/m^2^ for all conditions. To achieve this, illuminant intensities were set to 25.1 cd/m^2^, 25.4 cd/m^2^, and 25.6 cd/m^2^ for the natural, red-increased, and blue-increased distributions, respectively, in the 4000 K condition. For 6500 K, they were 26.3 cd/m^2^, 26.9 cd/m^2^, and 27.3 cd/m^2^, respectively. For 10,000 K, we used 27.4 cd/m^2^, 27.7 cd/m^2^, and 27.8 cd/m^2^, respectively.

### Results and discussion

**Figure 17.**
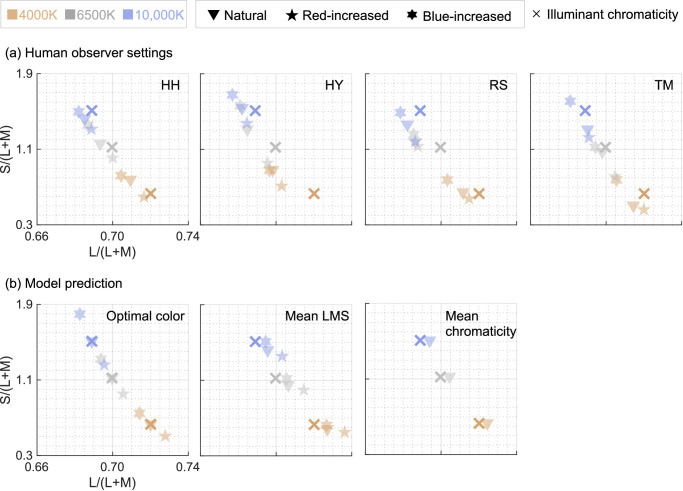
(a) Observers’ chromatic settings for each condition in Experiment 3. Different panels indicate a different observer. (b) Model prediction by the optimal color model, mean LMS model, and mean chromaticity model. The mean chromaticity model predicts the same chromaticity for any distribution condition by design; thus, only the prediction for the natural condition is shown here.

Figure 17 shows averages of the human observers’ settings across 20 settings for each condition. The presentation of results follows previous experiments.

In this experiment, Figure 17b is particularly important, as the optimal color model provided different chromaticities for each distribution condition by design. We see here that the prediction by the mean LMS model was also influenced by the shape of the distribution. The mean chromaticity model predicted the same chromaticity for any distribution condition; thus, any shift of the observers’ perceptual white point depending on distribution condition cannot be explained by the mean chromaticity model.

Figure 17a shows that all observers’ settings exhibited a systematic pattern. For the red-increased distribution, observers’ settings shifted in a higher L/(L+M) and lower S/(L+M) direction compared to settings under the natural distribution. For the blue-increased distribution, the observers’ settings shifted in a lower L/(L+M) and higher S/(L+M) direction. Thus, the shape of the color distribution had a strong effect on observers’ settings in this experiment. Importantly these shifts closely resemble the prediction pattern of the optimal color model, as shown in Figure 17b. These results would support our hypothesis that human observers use the shape of a color distribution to infer the chromaticity of an illuminant. However, note that the mean LMS model also seemed to predict shifts in the observers’ settings reasonably well. Later in this section, we quantify the degree of agreement between observers’ settings and model predictions based on correlation coefficient and RMSE values.

**Figure 18.**
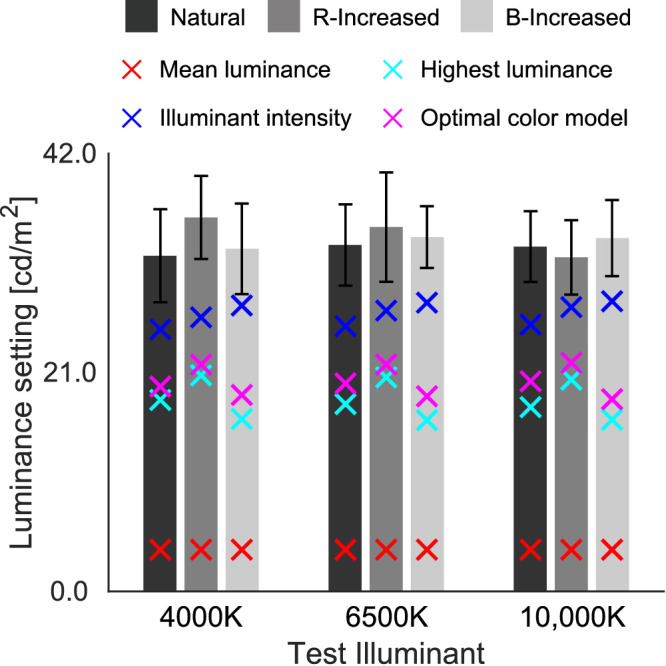
Observers’ luminance settings for each condition in Experiment 3. The red and cyan crosses indicate the mean luminance and highest luminance across 60 surrounding surfaces, respectively. The blue cross symbol shows the intensity of the test illuminant. The magenta cross denotes the prediction by the optimal color model. Error bars are ±*SE* across four observers.

Figure 18 shows the luminance settings for each condition. Overall, it can be seen that the observers’ luminance settings are closer to the illuminant intensity (i.e., ground truth) compared to Experiments 1 and 2 (Figures 6 and 13), suggesting better estimation of the intensity of illuminants. We performed two-way, repeated-measures ANOVAs with distribution condition (natural, red-increased, or blue-increased) and illuminant condition (4000 K, 6500 K, or 10,000 K) as the within-subject factors for the luminance settings. The main effects of distribution condition and illuminant condition were not significant: *F*(2, 6) = 0.149, *p* > 0.05; *F*(2, 6) = 4.06, *p* > 0.05, respectively. The interaction between two factors also was not significant, *F*(4, 12) = 1.32, *p* > 0.05. Thus, we found no significant difference for any pair of conditions in Experiment 3.

We again calculated correlation coefficients for the averaged observers’ settings (nine points) and each luminance statistic. Correlation coefficients were 0.2154 (*p* > 0.05), 0.3255 (*p* > 0.05), 0.1833 (*p* > 0.05), and 0.2701 (*p* > 0.05) for mean luminance, highest luminance, illuminant intensity, and optimal color model, respectively. Thus, none of the tested models here shows a particularly high correlation. This result is in agreement with Experiment 2.

**Figure 19.**
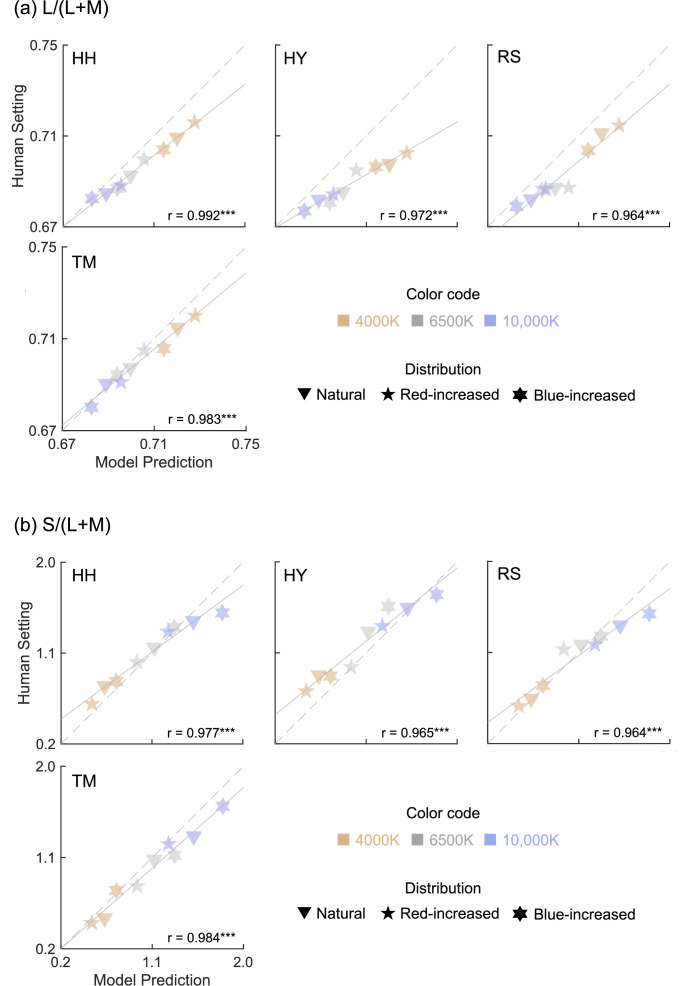
(a) Optimal color model prediction (horizontal axis) versus human observers’ settings (vertical axis) for the L/(L+M) direction. Different subpanels indicate different observers. The right-bottom numbers show correlation coefficient and *** indicates *p* values less than 0.001. The dotted straight line represents a unity line, and the solid line indicates a straight line fit to the data points. (b) The same plot for the S/(L+M) direction.

Because the main focus of this experiment was to examine the effect of distribution shape on observers’ settings, we do not show CIs that focused on the effect of illuminant change on observers’ settings. Instead, we examine the degree of agreement between model prediction and observers’ settings using correlation coefficients. Figure 19 shows a plot where the horizontal axis is the prediction of a optimal color model and the vertical axis is the observers’ settings in the L/(L+M) and S/(L+M) direction. The dotted line indicates a unity line. The solid line is a straight line fitted to the nine data points. The correlation coefficient is shown at the bottom right in each subpanel, where *** indicates a *p* value less than 0.001. The correlation coefficients show fairly high values, suggesting that the optimal color model can account for human observers’ behavior well.

These assessments allow us to identify the degree of correlation between model predictions and observers’ settings, but the data points should all fall on the unity line if the model prediction is perfect. For example, we see that data points are generally slightly off downward from the unity line for the L/(L+M) direction, especially for the 4000 K condition. To quantify the deviation of data points from the unity line, we calculated the RMSE values between model prediction and observer settings, across nine data points separately for the L/(L+M) and S/(L+M) directions. The correlation coefficients and RMSE together should allow us to reach a conclusion regarding the accuracy of model prediction.

**Figure 20.**
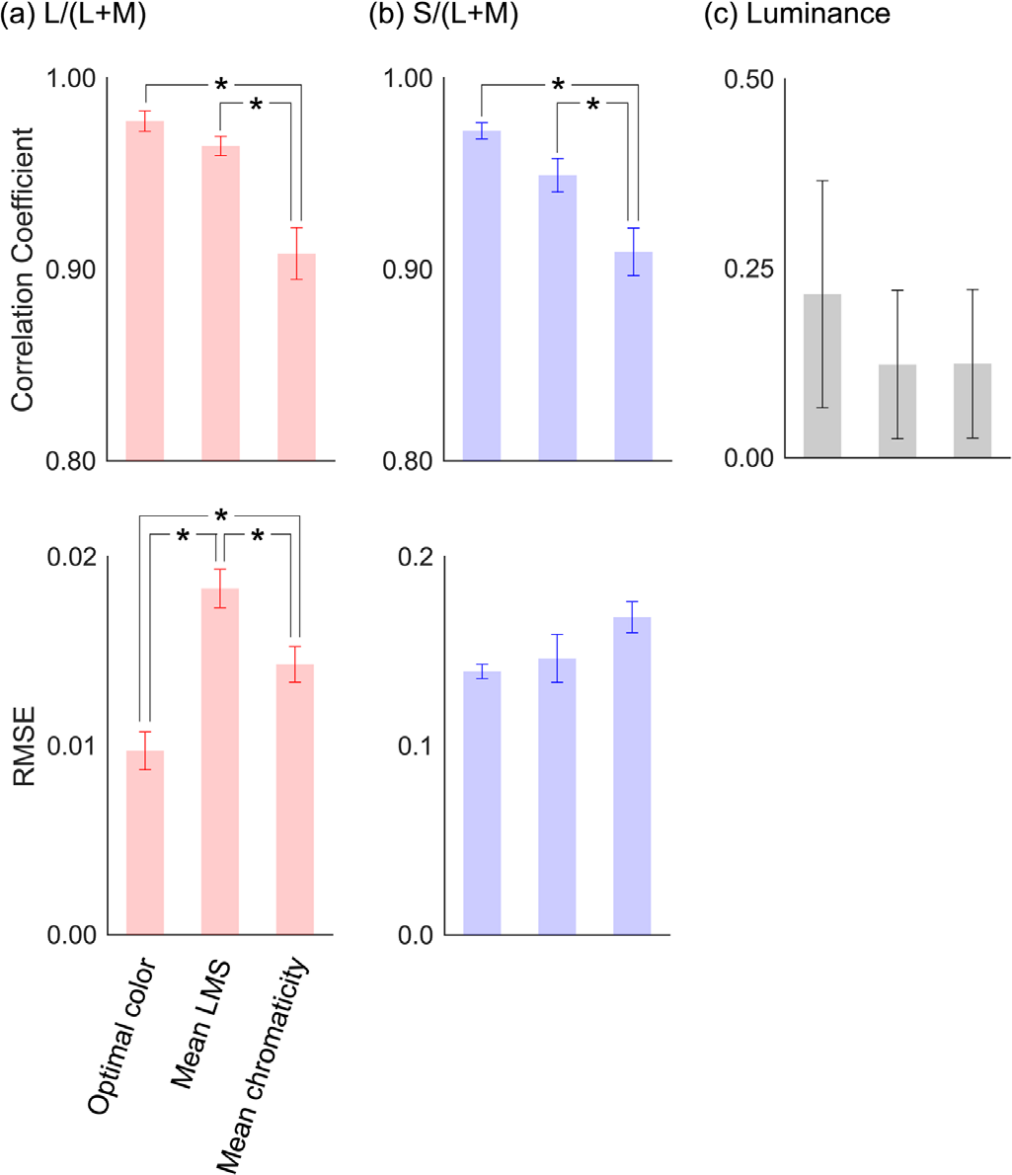
Correlation coefficient (upper panel) and RMSE (lower panel) between observers’ settings and model predictions for (a) L/(L+M), (b) S/(L+M), and (c) luminance. Values are averaged across four observers, and error bars indicate ± *SE* across four observers. Note that the range of the vertical axis is different for the luminance direction. Asterisks denote a significant difference (α < 0.05, Bonferroni's correction).

In Figure 20, we summarize correlation coefficients (upper subpanels) and RMSE values (lower subpanels) for the optimal color model and other models. We calculated both indices separately for each observer first (as demonstrated in Figure 19), and Figure 20 shows the averaged values across four observers. Here, the upper subpanels indicate that the optimal color model had the highest correlation coefficients for both chromatic channels. The mean LMS model shows a correlation close to that of the optimal color model. The vertical axis ranges between 0.80 and 1.00 for L/(L+M) and S/(L+M), indicating very high correlations for any tested model here. For luminance, the correlation coefficient is noticeably lower than L/(L+M) and S/(L+M) for any model. Also, variation among observers seems to be larger, as indicated by the length of the error bars. The mean LMS and mean chromaticity models gave the same estimation of illuminant intensity; thus, the correlation coefficients are also exactly the same. Regarding RMSE values, the optimal color model showed a lower value for L/(L+M) than did the other two models. Also, the trend held for the S/(L+M) direction, but the differences across models seem to be small. We do not show RMSE values for the luminance condition, as we believe that the absolute prediction from the mean LMS and mean chromaticity models should not be expected to match human observers’ luminance settings and that only a relative measurement matters here (e.g., correlation coefficient).

For correlation coefficients, we performed one-way, repeated-measures ANOVAs separately for each channel—L/(L+M), S/(L+M), and luminance—with model type (optimal color, mean LMS, and mean chromaticity models) as the within-subject factor. The main effect of model type was significant for L/(L+M) and S/(L+M), *F*(2, 6) = 15.8, *p* = .00408; *F*(2, 6) = 21.0, *p* = 0.00195, respectively, but not for luminance, *F*(2, 6) = 0.0094, *p* > 0.05. We next performed multiple comparison using Bonferroni's correction (significance level, 0.05). Pairs that showed significant differences are indicated by asterisks in Figure 20.

Also, for RMSE values, one-way, repeated-measures ANOVAs were conducted separately for L/(L+M) and S/(L+M) channels with model type (optimal color, mean LMS, and mean chromaticity) as the within-subject factor. The main effect of model type was significant for L/(L+M), *F*(2, 6) = 1687.0, *p* < 0.00001, but not for S/(L+M), *F*(2, 6) = 1.44, *p* > 0.05. We next performed multiple comparisons using Bonferroni's correction (significance level, 0.05); pairs that showed a significant difference are indicated by asterisks in Figure 20.

Overall, these results suggest that the optimal color model is better than either the mean LMS model or the mean chromaticity model in predicting observers’ estimations of L/(L+M) and that tested models are equally good at predicting observers’ estimations of S/(L+M). Also, in terms of predicting intensity estimation, none of tested models here was particularly good.

In summary, in Experiment 3, a proposed optimal color model accounted for the shift of chromatic settings in response to the change of the scene color distribution with high precision. Here, we note that our experiment still should not rule out the possibility of use by the visual system of the mean LMS model or any other models that we did not test in the present study. Nevertheless, empirical data here suggest that this geometry-based estimation of illumination might be a plausible algorithm for our visual system to infer the influence of illumination.

## General discussion

From the set of colors in a scene, how can we infer the color of the illuminants projecting onto the surface? An intuitive answer would be to use the chromaticities of surfaces (for example, when the illuminant is reddish, chromaticities of all surface become reddish). What is less known is that the luminance distribution also systematically changes, providing a diagnostic cue to the illuminant color. Our present study addressed the importance of the luminance distribution and specifically investigated whether subjective white-point settings recorded from human observers can be predicted by the optimal color model, which estimates illuminant color based on the shape of the color distribution. We conducted three psychophysical experiments to test this hypothesis. Experiments 1 and 2 were designed so that the optimal color model predicted a constant illuminant despite changes in the distribution shapes, and observers’ behavior generally agreed with this model prediction. In Experiment 3, we manipulated the shapes of the color distribution so that the optimal color model predicted the success or failure of color constancy, and we found that observers’ chromaticity settings followed the prediction well. These empirical data collectively suggest that the optimal color model could be a good candidate model for human color constancy, especially in estimating the chromaticity of illuminants in scenes that contain natural objects.

However, the accuracy of the model with regard to observers’ illuminant intensity estimations was largely limited. One notable feature in the results is that the observers’ luminance settings were substantially higher than the actual illuminant intensity (shown in Figures 6, 13, and 18). This implies that the human observers assumed that the presented surrounding stimuli were darker than the optimal colors, because under the estimated illuminant intensities the presented stimuli were not optimal colors. One might argue that because we know that white is very bright, observers simply increased the luminance of the test field up to the upper limit allowed by our experimental display. However, we confirmed that no observer settings reached the upper limit. If we think that optimal colors do not exist in the real world, it makes sense that observers assumed high intensity of the illuminant rather than high surface spectral reflectance. In the framework of the proposed model, there is a strong association between accuracies in illuminant intensity estimation and in color temperature estimation. To demonstrate this, let us consider a scene illuminated by a bluish illuminant. Under the bluish illuminant, bluish surfaces should become lighter, and one would expect that optimal color distribution under a reddish illuminant would be inappropriate, as it cannot cover light blue surfaces. However, this is true only when the illuminant intensity is properly estimated. In theory, if we assume a very intense illuminant, the optimal color distribution of any color temperature should cover the blue surface. In other words, for the model to estimate the color temperature precisely the illuminant intensity must be estimated well, too. Thus, inaccurate illuminant intensity estimation by human observers could be one reason for imperfect agreement between model prediction and observer settings. This conclusion is also supported by the finding that observers’ estimations of illuminant intensity were better in Experiment 3.

It should be noted that the present study used flat and matte experimental stimuli that are essentially an array of colors to eliminate other cues regarding the illuminant, such as the influence of memory color (Olkkonen, Hansen, & Gegenfurtner, 2008). In contrast, objects in the real world are usually three dimensional and sometimes contain specular reflection. Thus, a single object typically exhibits a color variation over a surface that may provide additional cues to color constancy. The use of simple stimuli may be a potential reason why we found a relatively low degree of color constancy (around 50%) in Experiments 1 and 2. Especially, the use of three-dimensional stimuli (Morimoto, Mizokami, Yaguchi, & Buck, 2017; Mizokami, 2019) and the presence of specular reflection (Yang & Shevell, 2002; Lee & Smithson, 2016) are claimed to be important for color constancy. However, it is also important to note that perfect color constancy is usually not observed in lab-based experiments, even when those cues are present. Logvinenko, Funt, Mirzaei, and Tokunaga (2015) also argued that perfect color constancy is, in theory, impossible because of the presence of illuminant metamerism (referred to as “metamer mismatching”). The purpose of the present study was to test whether human observers are able to maintain color constancy based on the shape of color distribution; thus, it was a necessary choice to keep our experimental stimuli as simple as possible to exclude the influence from other cues. In any case, it will be important to test how well our model agrees with human behavior in the presence of other cues.

In addition to the simplification of surface properties, there are at least two limitations regarding illuminant properties in this study. First, our test illuminants always changed along the blue–yellow direction. It would be interesting to test whether human color constancy still holds for non-black-body locus illuminants (e.g., magenta illuminant) to which we are not exposed in daily life. Interestingly, Delahunt and Brainard (2004) showed that our color constancy is not impaired under atypical illuminants, suggesting that our internal assumption about illuminant color is not constrained along the blue–yellow axis. Second, all experimental scenes were uniformly illuminated by a single illumination. In contrast, objects placed in the real world tend to receive incident light from every direction, and the spectral distribution of this light may change from one direction to another. For example, in sunny outdoor scenes, light from above tends to come from sunlight or a skylight, but the object also receives light from below dominated by a secondary reflection from other objects in the scene. Recent studies indeed have shown that natural scenes have a significant amount of directional spectral variation (Morimoto, Kishigami, Linhares, Nascimento, & Smithson, 2019). For a scene illuminated by a single light source, as shown in Figure 1, a single optimal color distribution shows a complete representation of the surface color gamut; however, for a scene under multiple illuminants, the gamut accordingly expands. For this reason, our model also must consider more than one optimal color distribution. In general, estimation of multiple illuminations increases the complexity of the color constancy problem and thereby inflates computational cost; therefore, our model may also suffer because there are too many combinations of optimal color distributions to fit. In recent years, a growing body of research has investigated the influence of directional-dependent illumination on human color constancy or other functions of color vision (Fleming, Dror, & Adelson, 2003; Doerschner, Boyaci, & Maloney, 2004; Morimoto & Smithson, 2018). Our present study suggests that the optimal-color-based explanation works reasonably well when there is only one illuminant in a scene. Testing the limitation of human color constancy under multiple illuminants allows us to investigate the degree to which our proposed model can be applied.

Our model has some parallels with the idea that the visual system uses statistical regularities hidden in natural scenes which can act as internal references to calibrate our perception (Gilchrist et al., 1999; Lee & Smithson, 2012). For example, our white-point settings when no context is presented seem to spread along the chromatic variation of natural scenes, implying mechanisms to normalize color appearance to scene statistics (Bosten, Beer, & MacLeod, 2015). Empirical data also suggest the influence of natural scene statistics on color vision mechanisms such as chromatic adaptation, color discrimination, and color constancy (Webster & Mollon, 1997; MacLeod, 2003; Webster 2011). Moreover, there is evidence that our unique hue points can be recalibrated following exposure to manipulated chromatic signals (Neitz, Carroll, Yamauchi, Neitz, & Williams, 2002) or the seasonal change of chromatic statistics (Welbourne, Morland, & Wade, 2015). DeLawyer, Tayon, Yu, and Buck (2018) showed that the amount of S-cone excitation could be a clue as to whether a color change stems from an illuminant change or a surface change.

We have suggested that the optimal color model explained observers’ settings reasonably well, and the degree of agreement typically outperformed two simple alternative statistical models based on mean chromaticity or mean LMS. However, the prediction accuracy of our model was not perfect under any of the conditions. It is possible that the observers used a more complicated strategy for illuminant estimation, and one might argue that more sophisticated computational color constancy algorithms could better explain the observers’ behaviors. As briefly reviewed in the Introduction, a number of machine vision algorithms have been developed, and some aspects of them are similar to those of our proposed model. This study did not make an extensive comparison of performance across computational algorithms for the following two reasons. First, such algorithms normally operate in the RGB color space and thus cannot be directly applied to our experimental stimuli, which were defined in terms of the proportion of cone excitations: L/(L+M), S/(L+M), and L+M. Second, and more importantly, they were designed to solve the machine vision task to eliminate the illuminant influence from a given image, and they do not necessarily have a direct link to the mechanisms that underlie human color constancy. For example, it is normally not considered whether calculations are feasible for visual system; consequently, signals to which our visual system would not have access are often used. Hence, we figured that making such a comparison digresses from the purpose of the present study. However, research comparing human vision and machine vision is attracting increasing attention (Geirhos, Meding, & Wichmann, 2020) and might lead to the development of human-like algorithms that operate based on sensory signals in visual pathways.

Major findings in the present study are that we can account for human observers’ estimation of illumination if we assume that the visual system has full access to the distribution of optimal colors. However, how valid are these assumptions? How plausible is it for us to determine the physical limit of colors? It remains an open question as to whether human observers are able to access the shape of the optimal color distribution by observing statistical regularities in natural scenes. Figure 1 shows that when we plot 49,667 natural objects at once, it very much resembles the shape of the optimal color distribution. This indicates that if we integrate the luminance versus chromaticity distribution over a long period, it may be possible for us to learn where the physical limits are. We believe that the observer's internal assumption about the physical limits of surface colors can be visualized by measuring luminosity thresholds over various chromaticities. Our earlier data (Uchikawa, Koida, Meguro, Yamauchi, & Kuriki, 2001; Uchikawa, Fukuda, & Morimoto, 2017) indicate that the locus of luminosity thresholds resembles the luminance of optimal colors. Additionally, Speigle and Brainard (1996) suggested that it is possible to relate luminosity thresholds to the upper luminance boundary estimated from the luminance distributions of natural objects, which agrees with the optimal color distribution as shown in Figure 1. Recent studies also have begun to show some success with such a learning-based approach in other research domains (Fleming & Storrs, 2019). For complex functions such as color constancy, where complete implementation requires solving inverse optics, a learning-based strategy provides a powerful alternative. Thus, it may be plausible for our visual system to learn and utilize optimal color distributions to implement a mathematically challenging constancy mechanism. The extension of research in this direction might yield exciting insights into long-standing questions regarding the field of color vision.

## Supplementary Material

Supplement 1
