# Supplementary material for: Human color constancy based on the geometry of color distributions
Source: J Vis. 2021 Mar 4;21(3):7. doi: 10.1167/jov.21.3.7 (PMC7937993; doi:10.1167/jov.21.3.7)
Supplement: Supplement 1 [file jovi-21-3-7_s001.docx]

**Supplementary Material**

In this study, we introduce constancy index defined in equation (2) that considers both the distance and the angular deviation between the observer’s setting and the ideal match. However, it is not the only way to define a constancy index. There are indeed two frequently used indices in behavioural color constancy research that are determined purely by the distance: (i) constancy index CI (Arend et al., 1991) and (ii) Brunswick Ratio BR (Troost and de Weert, 1991). In order to check that our main conclusions hold even if we use these distance-based metrics, we calculated CI_Arend_ and BR for our data. Figure S1 shows how to calculate these two commonly used indices. One feature in our experimental data was that observers’ settings under 6500K did not match the chromaticity of 6500K. We believe this is due to individual criterion differences of white point because we did not provide extremal references to observers during experiments. These biases in the reference white need to be corrected before calculating constancy indices. Thus, we first shifted the settings under 3000K and 20000K in a direction to cancel out these internal biases as shown in the left part of Figure S1. Then, using the shifted chromaticity under a test illuminant (20000K in this case), we calculated *CI_Arend_* and *BR* based on following equations.

${CI}_{Arend}= 1-\frac{b}{a}$ ・・・　(S1)

$BR= \frac{c}{a}$ ・・・　(S2)

Figure S1**:** Cancellation of the bias induced by observer’s internal criterion differences (left part) and the definition of distances a, b and c used for the calculation of constancy indices (right part). Past constancy indices were defined as CI_Arend_ = 1 – b/a, BR = c/a.

The resultant values are summarized in Table 1 where each cell shows mean index value±S.E. across 4 observers. It is clear that values are highly consistent across indices. We speculate that this is because angular deviation (θ as defined in Figure 7) in our data was relatively small. In any case, these calculations confirmed that our finding holds even if we used other commonly used indices.

Table S1: Summary of the constancy index proposed in the present study and other constancy indices. Each cell indicates mean ± S.E. across 4 observers.
